# Signals of Variation in Human Mutation Rate at Multiple Levels of Sequence Context

**DOI:** 10.1093/molbev/msz023

**Published:** 2019-02-07

**Authors:** Rachael C Aikens, Kelsey E Johnson, Benjamin F Voight

**Affiliations:** 1 Program in Biomedical Informatics, Stanford University School of Medicine, Stanford University, Stanford, CA; 2 Genetics and Epigenetics Program, Cell and Molecular Biology Graduate Group, Perelman School of Medicine, University of Pennsylvania, Philadelphia, PA; 3 Department of Systems Pharmacology and Translational Therapeutics, Perelman School of Medicine, University of Pennsylvania, Philadelphia, PA; 4 Department of Genetics, Perelman School of Medicine, University of Pennsylvania, Philadelphia, PA; 5 Institute for Translational Medicine and Therapeutics, University of Pennsylvania, Philadelphia, PA

**Keywords:** mutation rate, statistical genetics, sequence context models

## Abstract

Our understanding of the human mutation rate helps us build evolutionary models and interpret patterns of genetic variation observed in human populations. Recent work indicates that the frequencies of specific polymorphism types have been elevated in Europe, and that many more, subtler signatures of global polymorphism variation may yet remain unidentified. Here, we present an analysis of the 1000 Genomes Project supported by analysis in the Simons Genome Diversity Panel, suggesting additional putative signatures of mutation rate variation across populations and the extent to which they are shaped by local sequence context. First, we compiled a list of the most significantly variable polymorphism types in a cross-continental statistical test. Clustering polymorphisms together, we observe three sets that showed distinct shared patterns of relative enrichment among ancestral populations, and we characterize each one of these putative “signatures” of polymorphism variation. For three of these signatures, we found that a single flanking base pair of sequence context was sufficient to determine the majority of enrichment or depletion of a polymorphism type. However, local genetic context up to 2–3 bp away contributes additional variability and may help to interpret a previously noted enrichment of certain polymorphism types in some East Asian groups. Moreover, considering broader local genetic context highlights patterns of polymorphism variation, which were not captured by previous approaches. Building our understanding of mutation rate in this way can help us to construct more accurate evolutionary models and better understand the mechanisms that underlie genetic change.

## Introduction

The process of mutation is a formative force in molecular evolution because it generates the genetic variation that can be acted upon by natural selection. Quantitative and qualitative insights regarding the mutation rate in human populations can facilitate the construction of increasingly informative models of human evolutionary history, targets of natural selection, and perhaps even genetic or environmental mechanisms that confer genomic stability and drive genetic change. Although the rates of DNA mutation and repair have been known to differ widely between certain individuals (Conrad et al. 2011), within and between chromosomes (Hodgkinson and Eyre-Walker 2011), and down to specific local sequences (Aggarwala and Voight 2016), the biological mechanisms underlying mutation rate variability across the genome are not completely understood. Recent work has suggested that the mutation rate in humans may have been in flux over recent evolutionary history (Harris 2015; Harris and Pritchard 2017; Mathieson et al. 2017). A key observation supporting this hypothesis is that the relative proportions of certain types of polymorphisms vary across populations. Most notably, studies cite the strong enrichment of C/T polymorphisms at certain trinucleotide contexts in Europe and South Asia (Harris 2015; Harris and Pritchard 2017; Mathieson et al. 2017). These reports have also documented additional polymorphism types that appear to vary in frequency across populations (Harris and Pritchard 2017; Mathieson et al. 2017).

However, there are two lines of inquiry that have not yet been fully explored in these data. First, because clusters of polymorphisms with similar global profiles of enrichment might be driven by a shared mechanism, it is worthwhile to ask not only which polymorphism types vary across the globe but also how variable polymorphism types group together as putative “signatures” of mutation rate variations. These signatures can then be analyzed together, for example, by searching for “pulses” of increased polymorphism volume across the site frequency spectrum which may indicate the timing of mutation rate changes in the distant past (Harris and Pritchard 2017). Developing a better understanding of these signatures of polymorphism variation may help to link such changes to a putative genetic or environmental cause. Second, models of local genetic sequence context beyond a single flanking nucleotide have not yet been applied to the study of variability in mutation rates across populations. Our previous work has demonstrated that windows of flanking genetic sequence broader than the trinucleotide context may uncover additional detail in mutation rate variability across the genome (Aggarwala and Voight 2016; Zhu et al. 2017). The consideration of greater numbers of upstream and downstream base pairs of genetic context could help to interpret the mechanism by which mutation rate varies across populations. For example, strong effects stemming from more remote sequence context may drive signals of mutation rate variation observed in a lower order (i.e., trinucleotide) context, indicating that the underlying mechanism may rely on the broader local nucleotide configuration. As such, models that consider broader windows of local context may highlight subtle variation in polymorphism that might not have otherwise been detected.

For these reasons, we sought to expand upon previous studies by identifying polymorphisms at multiple context levels and quantifying how they vary across populations. To this end, we have applied a combination of sequence context frameworks to analyze the current release of the 1,000 Genomes Project, spanning >2,000 subjects, and, where possible, corroborative support from the Simons Diversity Genome Panel. Herein, we aimed to catalog population-level heterogeneity in polymorphism across the spectrum of sequence contexts, and cluster variable polymorphism classes that we observe into common mutational signatures which can be analyzed together across populations.

## Results

To quantify differences in the frequencies of mutation types across populations, we assembled sets of genetic variants specific to Africans, Europeans, South Asians, and East Asians (504, 503, 489, and 504 individuals, respectively, excluding recently admixed American populations) from the 1000 Genomes Project, phase 3 (1KG) (Auton et al. 2015). As genetic variants in the coding genome are likely to be under purifying selection, we focused on variants observed in the noncoding genome to minimize the influence of selective pressures (see Materials and Methods). Our final sets consisted of 7 million, 1.2 million, 1.96 million, and 1.99 million variants private to African, European, East Asian, and South Asian populations, respectively. From this point on, we use the notation X_1_→X_2_ to represent a polymorphic sequence in the human genome with reference allele X_1_ and alternate allele X_2_. Additionally, NX_1_N→X_2_ denotes an X_1_→X_2_ polymorphic site as before where the original surrounding reference sequence was NX_1_N (e.g., CAG→T, which denotes an A/T polymorphism with flanking bases C and G).

In addition, we performed replication analyses using the analogous populations from the Simons Genome Diversity Project (SGDP) (Mallick et al. 2016), publicly available through the Seven Bridges Cancer Genomics Cloud data portal. We used a subset of the data set which consisted of 44 Africans, 69 West Eurasians (used as a comparison to the “European” population from 1KG), 47 East Asians, and 39 South Asians. After preprocessing (see Materials and Methods), this amounted to a replication data set of 3.6 million, 0.7 million, 0.5 million, and 0.3 million variants private to African, European, East Asian, and South Asian populations, respectively (supplementary table 1, Supplementary Material online).

### Identifying Novel 3-mer Polymorphism Classes That Vary across Ancestral Groups

We first compiled a list of polymorphisms in trinucleotide (i.e., “3-mer”) contexts that appear heterogeneous in their representation across the globe. To this end, we extended a previously described (Harris 2015; Harris and Pritchard 2017) approach for comparing counts of polymorphisms between pairs of populations (see Materials and Methods). Rather than making all possible pairwise comparisons between ancestral groups for a given polymorphism class, we perform a single test of homogeneity in private polymorphism for that class across Africa, Europe, East Asia, and South Asia. In addition to reducing the required number of hypothesis tests compared with previous methods (important later for analyses with broader windows of sequence context), this statistical framework allowed us to rank order polymorphism types by the significance of their variation across all ancestral groups. After replicating previous results (Harris 2015) as a technical control (supplementary fig. 1, Supplementary Material online), we applied our test to each 3-mer polymorphism type (96 total), applying a modified *P*-value correction (*P*_ordered_) as previously described (Harris and Pritchard 2017), in addition to Bonferroni correction for multiple hypothesis testing (nominal significance threshold *P*_ordered_ < 5 × 10^−4^, see also Materials and Methods).

As expected, the most compelling group of polymorphisms was composed of C→T polymorphism classes previously reported to be enriched in Europe and South Asia (table 1). All four types that have been previously noted as part of this signal: TCC→T, ACC→T, TCT→T, and CCC→T were among the six most variable polymorphisms (all *P*_ordered_ < 1 × 10^−68^).

**Table 1.** 3-Mer Polymorphism Classes Most Highly Significantly Variable across Ancestral Groups.

Notes	3-Mer	Relative Rate in Africa^a^^,^^b^	Relative Rate in Europe^a^	Relative Rate in South Asia^a^	Relative Rate in East Asia^a^	*P*_ordered_^b^ (1KG)	*P*_ordered_^b^ (SGDP)
Previously reported C→T elevation in Europe (Harris 2015; Harris and Pritchard 2017; Mathieson et al. 2017)	TCC→T	1	*1.56*	*1.20*	1.00	≈ 0	≈ 0
	ACC→T	1	*1.20*	*1.07*	*0.93*	3 × 10^−308^	3 × 10^−161^
	TCT→T	1	*1.17*	*1.06*	0.98	3 × 10^−196^	≈ 0
	CCC→T	1	*1.06*	*1.03*	*0.95*	3 × 10^−69^	7 × 10^−68^
Not previously highlighted	GAT→T	1	*1.06*	*1.13*	*1.21*	6 × 10^−111^	2 × 10^−23^
	ACC→A	1	*1.04*	*1.10*	*1.15*	2 × 10^−98^	5 × 10^−17^
	ACA→T	1	*0.95*	0.97	*0.93*	3 × 10^−60^	3 × 10^−30^
	TCA→T	1	*1.06*	*1.03*	0.97	1 × 10^−53^	2 × 10^−13^
	ACT→T	1	1.02	1.00	*0.94*	4 × 10^−51^	2 × 10^−45^
	GAC→T	1	1.02	*1.09*	*1.20*	7 × 10^−40^	6 × 10^−10^
	GCC→T	1	*1.06*	1.03	1.02	2 × 10^−37^	4 × 10^−14^
	CAC→G	1	*0.93*	*0.92*	*0.96*	8 × 10^−33^	5 × 10^−16^
	CCA→T	1	*0.93*	*0.97*	*0.95*	4 × 10^−27^	2 × 10^−21^
	GCC→A	1	1.02	*1.07*	*1.08*	9 × 10^−25^	3 × 10^−5^

Note.—Italic numbers indicate a significant difference in polymorphism proportion compared with Africa (*P* < 1 × 10^−5^) in a pairwise chi-square test using the *P*_ordered_ correction procedure as previously described (Harris and Pritchard 2017) (see also Materials and Methods).

a Approximate private mutation rates (per generation per site) for each continent were inferred by normalizing estimated polymorphism probabilities using all private mutations to the de novo mutation rate estimated from Kong et al. (2012), and then subsequently normalized relative to inferred rate in Africa.

b *P*-values apply the *P*_ordered_ correction procedure (Harris and Pritchard 2017) (see also Materials and Methods).

We further observed that all four CpG transition mutations were variable between populations (*P*_ordered_ < 1 × 10^−31^). However, our analysis in SGDP populations revealed that patterns of CpG enrichment were not consistent between these two data sets (supplementary fig. 2, Supplementary Material online), which was previously observed (Harris and Pritchard 2017), and one further report has noted that CpG transitions may vary between populations due to recent demographic history rather than mutation rate differences (Mathieson et al. 2017). As a result, we restricted our subsequent analyses to non-CpG polymorphism classes.

We next examined the remaining variable polymorphisms for novel signatures of mutation rate variation. We found that 63 of the 96 possible 3-mer types exceeded our Bonferroni correction for multiple tests across ancestral groups (supplementary table 2*A*, Supplementary Material online), and an overlapping set of sixty-seven 3-mer types exceeded Bonferroni correction under the same testing procedure in SGDP (supplementary table 3, Supplementary Material online). Therefore, we opted to consider only polymorphism classes which 1) were significant at *P*_ordered_ < 5 × 10^−4^ in both 1KG and SGDP and 2) showed acceptable agreement in their patterns of variation between data sets (supplementary table 4, Supplementary Material online). The 14 most heterogeneous such polymorphism types based on their significance in 1KG (corresponding to a *P*_ordered_ < 1 × 10^−25^) are shown in table 1. To facilitate comparison of the relative enrichment between continental groups, we used all private polymorphisms to infer a mutation rate for each 3-mer (per generation per site) normalized to the average estimated de novo mutation rate from Kong et. al (Kong et al. 2012) (see Materials and Methods, supplementary figs. 3 and 4, Supplementary Material online). There are many reasons to expect that the exact numeric estimates of private mutation rate do not agree between SGDP and 1KG, for example, due to sequencing artifacts or sampling differences between the two data sets. As a result, “agreement” between the data sets was determined based on the relative enrichment and depletion of polymorphisms across continents, rather than the absolute mutation rate estimates.

In addition to the C→T polymorphisms mentioned above, we observed several additional polymorphism classes in our top ranked replicated set that have not yet been specifically noted in previous studies (table 1). One polymorphism, TCA→T, showed enrichment in Europe and South Asia, a profile similar to the previously noted C→T polymorphism types elevated in Europeans (table 1). The pattern of enrichment for ACT→T was similar to this, with greatest representation in Europe and South Asia, but with a higher proportion in Africa compared with other polymorphism types from this group. A final polymorphism, GCC→T, was likewise enriched in Europe and South Asia, but also showed an elevation in East Asia.

Interestingly, GAT→T, ACC→A, GAC→T, and GCC→A polymorphisms displayed a similar profile of heterogeneity distinct from previously reported signatures of variation: the highest rates in East and South Asia, with intermediate levels in Europe, relative to Africa. This suggests that these three polymorphism types may represent a group of polymorphisms enriched in Asia. The remaining three types, ACA→T, CAC→G, and CCA→T, were most elevated in Africans, with lower rates in East and South Asians and Europeans, perhaps suggestive of one or more Africa-enriched polymorphism groups.

As an additional validation in 1KG, we estimated the mutation rate separately on each chromosome and found that these patterns of enrichment hold relatively consistently across the genome for each of these polymorphism types (supplementary fig. 5, Supplementary Material online). Taken together, these results support the proposition that there may be several previously unreported signatures of variation in mutation rate observed at the 3-mer scale, beyond the previously reported signal of European C→T enrichment.

### Hierarchical Clustering of 3-mer Mutational Signatures

We next sought to identify sets of polymorphism types that share similar patterns of enrichment or depletion across the globe, which we hypothesize might be influenced by a common underlying mechanism. To this end, we performed hierarchical clustering and visual inspection of 3-mer polymorphism types based upon their relative inferred mutation rates in each of the twenty 1KG subpopulations comprising the nonadmixed continental groups from our initial analysis.

**Fig. 1.**
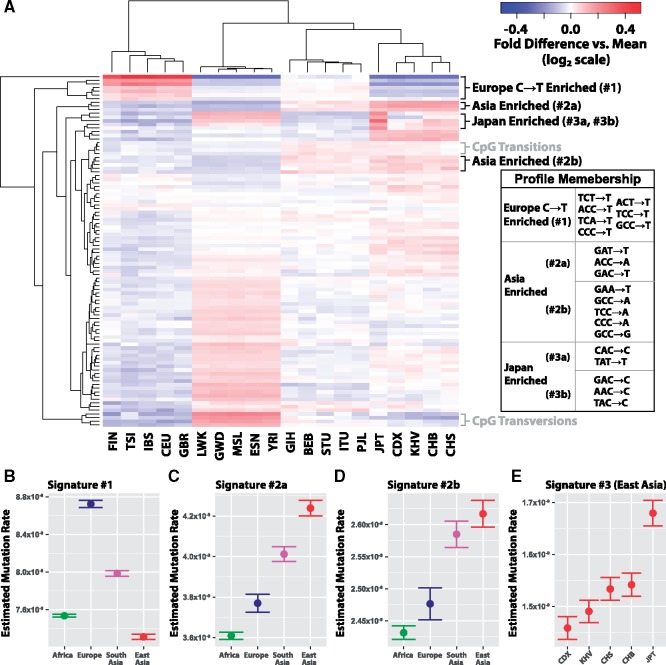
Signatures of mutation rate by trinucleotide context highlight variability across populations. (*A*) Heatmap of all 3-mer polymorphisms, clustered based on their relative rates in each of 20 nonadmixed 1000 Genomes Project populations. Clusters of interest are labeled, and their membership is detailed in the table to the right. Polymorphisms are clustered and colored based on fold elevation over the mean mutation rate for each mutation type. All units are log base 2 transformed, with red color corresponding to enrichment and blue to depletion. Population codes along the bottom correspond to the three-letter codes assigned in the 1000 Genomes data set. (*B*–*D*) Approximate 95% confidence interval estimates of inferred mutation rate across continental groups in 1KG for signatures #1, #2a, and #2b. (*E*) Inferred mutation rates for signature #3 shown across five East Asian subpopulations: Chinese Dai in Xishuangbanna (CDX), Vietnamese (KHV), Han Chinese from Beijing and Southern China (CHB and CHS), and Japanese in Tokyo (JPT).

We highlight three distinctive patterns of polymorphism rates—denoted here as “signatures”—that emerged from the clusters of 3-mer polymorphism types (fig. 1*A*). Signature #1 corresponds to an enrichment of C→T polymorphism in Europe, and includes all four 3-mers previously reported to comprise this signal (Harris and Pritchard 2017; Mathieson et al. 2017) (fig. 1*B*, replication in SGDP shown in supplementary fig. 6*A*, Supplementary Material online). The remaining three polymorphisms grouped with this signature, TCA→T, ACT→T, and GCC→T, are noted in the previous section (table 1), and represent variable polymorphisms not previously reported to be part of the Europe-enriched C→T signature. This signature appeared to be further broken into three subgroups in our clustering: TCC→T representing the strongest signal; then ACC→T, and TCT→T; and finally, CCC→T, TCA→T, ACT→T, and GCC→T.

The next signature (#2a) consists of GAT→T, ACC→A, and GAC→T, which are elevated in East and South Asia (fig. 1*C*). All three of these were noted above for their cross-continental heterogeneity and consistency across 1KG and SGDP (*P*_ordered_ < 7 × 10^−40^, table 1). An additional set of five polymorphisms (#2B) follows a similar pattern across continents (fig. 1*D*). These substitution classes, GCC→A, TCC→A, CCC→A, CCC→G, and GAA→T, are also significantly heterogenous across continents in both data sets and show consistent transcontinental patterns in 1KG and SGDP (table 1, supplementary table 4, and supplementary figs. 3, 6*B*, 6*C*, and 7, Supplementary Material online).

Next, we observed two clusters (signatures #3a and #3b) that appear enriched in Japan and other populations in East Asia, relative to other continental groups. This unit comprised the *AC→C polymorphisms, as well as TAT→T, corresponding with a previous report (Harris and Pritchard 2017) which documented that *AC→C, and TAT→T mutation types separate East Asians in a principal component analysis. Consistent with this previous observation, we find the same pattern of enrichment of signature #3 across East Asia (fig. 1*E*). However, given the relatively few individuals sampled within Asian subcontinental groups (i.e., Japan and Han China), we lacked statistical power to confirm this signature. Taken collectively, the polymorphisms comprising signatures #2 and #3 may represent two distinct patterns of enrichment for certain mutation types in Asia, although more work remains to be done to understand the natures of these variations.

We observed two additional clusters, both of which involve polymorphisms within CpG contexts, corresponding to either CpG transitions or transversions (fig. 1*A*). However, the proportions of the polymorphism types in these signatures do not appear to agree between the SGDP and 1KG datasets (supplementary figs. 2 and 8, Supplementary Material online). This suggests that these polymorphisms may be the results of an experimental artifact or some other aspect of population demography, rather than a true divergence in mutation rate. In sum, the clusters identified here highlight sets of polymorphisms whose relative proportions are similar across populations encompassed by the 1000 Genomes Project.

### Broader Sequence Contexts around 3-mer Signatures

**Fig. 2.**
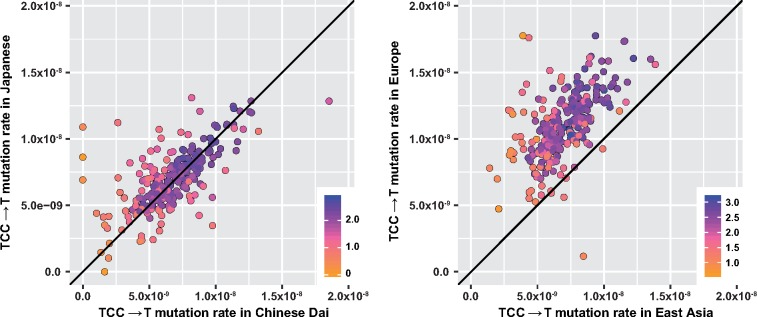
Mutational signatures driven at the 3-mer context level. Each point represents a 7-mer expansion of the 3-mer subtype shown, plotted based on its estimated mutation rate in 1KG within each of the two populations displayed. Colors indicate the log (base 10) of the number of polymorphisms observed for that 7-mer class. (*A*) When a 3-mer polymorphism type occurs at equal rates in two related populations, most of the 256 7-mer expansions of this 3-mer appear along the diagonal *y* = *x* line. (*B*) For TCC→T and the other C→T polymorphism types elevated in Europe, the bulk of the 7-mer expansions lie above the *y* = *x* diagonal, indicating that there has been a substantial difference in mutation rate between Europe and East Asia, and this difference is driven by effects at the 3-mer, rather than the 7-mer level.

Our previous work has indicated that flanking nucleotides within broader windows of sequence context around a genetic locus strongly correlate with the probability of polymorphism (Aggarwala and Voight 2016; Zhu et al. 2017). Thus, we next took each 3-mer type identified as heterogeneous in the previous section and measured the frequency of private polymorphisms in a broader window of local sequence context that considered three flanking nucleotides (i.e., a heptanucleotide, or “7-mer” context window). This subdivided each 3-mer polymorphism type into 256 distinct 7-mer classes, allowing us to ask whether the population-specific heterogeneity was common to all 7-mer expansions, or confined to a specific subset of those contexts. To this end, we plotted the relative inferred mutation rates of those polymorphisms in pairs of populations from 1KG. If there were no signal of mutation rate difference between populations, we would expect all 7-mer expansions to be distributed along the *y* = *x* diagonal (case I, e.g., fig. 2*A*). If the most important local features driving a mutational signal lay within a single-nucleotide base of the polymorphic locus, then we would expect all 7-mers to lie together off the diagonal (case II, e.g., fig. 2*B*). Alternatively, if a 3-mer signal were actually driven by a handful of highly variable 7-mer polymorphism types, only a handful of exceptional 7-mer types would lie far from the *y* = *x* line (case III).

**Fig. 3.**
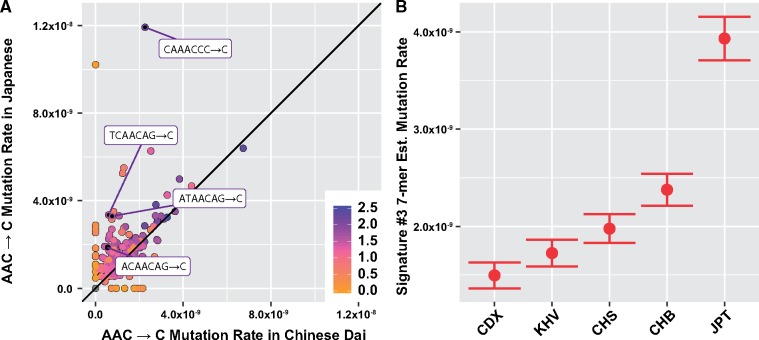
Patterns of variation within in East Asia among 7-mers from signature #3. (*A*) Most 7-mer expansions of AAC→C are the same in Chinese Dai versus Japanese, with the exception of some highly variable 7-mer polymorphism types. Polymorphisms significantly heterogeneous between Japan and Chinese Dai are labeled. (*B*) Estimated private mutation rate of the fourteen 7-mer polymorphism types shown in table 2 displayed across each East Asian subpopulation from 1KG. Brackets indicate approximate 95% confidence intervals.

We found that nearly all of the 3-mers comprising signatures #1 and #2 matched case II (fig. 2*B*, supplementary figs. 9 and 10, Supplementary Material online). Thus, the global variation in European C→T elevation, the CpG transitions, and the Asian signature #2 elevation was not clearly linked with sequence context features beyond a single flanking nucleotide base. However, the polymorphisms comprising signature #3 more closely matched case III, indicating that the Japanese enrichment of the *AC→C and TAT→T polymorphisms might be driven by a handful of 7-mer polymorphism types heterogeneous across East Asia (fig. 3, supplementary fig. 11, Supplementary Material online).

To explore this finding in more detail, we sought to identify the key 7-mer types underlying this 3-mer signature. To this end, we considered each of the 1,280 possible 7-mer expansions of *AC →C and TAT→T, testing for heterogeneity between Japanese individuals from Tokyo (JPT, higher signature #3 polymorphism proportion) and Chinese Dai from Xishuangbanna (CDX, lower signature #3 polymorphism proportion). We found fourteen 7-mer polymorphism types that were elevated in JPT relative to CDX (false discovery rate-adjusted *P* < 0.05; table 2, fig. 3*B*). Of these, ten have the shared motif NNNACAG→C. Curiously, we also observed that 4 out of the 14 polymorphism types were enriched on the X chromosome in East Asia, relative to the autosomes (though we note that many of the ten other polymorphism classes had too few observed polymorphisms on the X chromosome to justify a valid statistical test, see table 2). Due to the limitations in sample size (the corresponding populations are represented by five or fewer individuals), we did not attempt to replicate these analyses in SGDP.

**Table 2.** 7-Mer Polymorphisms from Signature #3 Which Are Heterogenous among East Asians.

7-Mer	FoldEnrichmentin Japan^a^	FDR-Adjusted *P*(enrichment in Japan)^b^	Expected Polymorphic Sites on X	Observed Polymorphic Sites on X	*P*(X enrichment)^c^
TTTATTT→T	2.14	6.4 × 10^−22^	48	65	0.009*
CAAACCC→C	5.28	1.3 × 10^−12^	6.2	27	6.7 × 10^−10^*
AGTACAG→C	16.3	1.3 × 10^−7^	3.0	4	—
TCAACAG→C	5.64	9 × 10^−4^	2.2	7	0.009*
ATAACAG→C	4.37	0.001	3.3	7	0.4
ATGACAG→C	4.62	0.001	2.4	4	—
CCCACAG→C	2.61	0.001	4.6	25	3.3 × 10^−11^*
ACCACCA→C	3.29	0.03	2.7	3	—
AAGACAG→C	3.20	0.03	3.1	4	—
AATACAG→C	3.20	0.03	2.8	1	—
ACAACAG→C	4.62	0.03	2.3	3	—
ATCACAG→C	2.80	0.03	3.1	4	—
GTGACAG→C	5.86	0.03	1.2	1	—
TTTATTA→T	1.63	0.04	13.1	15	0.3

a Fold increase in inferred mutation rate in Japanese in Tokyo compared with Chinese Dai in Xishuangbanna.

b Based on a chi-square test. Only polymorphisms with false discovery rate (FDR) < 0.05 are included.

c Enrichment on the X chromosome in East Asia was calculated according to a one-sided binomial test (see Materials and Methods). The significance values of tests for polymorphism classes with five or fewer observations on the X chromosome were not calculated.

* Nominal significance (*P* < 0.05).

### Variable Polymorphism Types among Broader Sequence Context Models

Motivated by this result and previous work (Aggarwala and Voight 2016), we next hypothesized that additional signals of mutation rate variation might be observable only in specific pentanucloetide (i.e., “5-mer”) or 7-mer polymorphism types. If this were true, considering a broader span of sequence context would highlight novel signals of mutation rate variation not evident from 3-mer level analyses. To this end, we applied the homogeneity-testing framework described above to each of the 1,536 possible 5-mers and the 24,576 possible 7-mer polymorphism types.

Within a 5-mer sequence context, we found that 156 out of 1,535 tested polymorphism types surpassed a Bonferroni-adjusted significance threshold (see Materials and Methods and supplementary table 2*B*, Supplementary Material online; one 5-mer polymorphism was not observed a sufficient number of times in each population to justify a valid statistical test). Of these, 57 represent expansions of Europe-elevated 3-mer polymorphisms from signature #1 (e.g., CTCCA→T, an expansion of the TCC→T 3-mer). An additional 28 represent expansions of 3-mer polymorphisms in signature #2. However, the remaining 71 significantly variable 5-mer polymorphisms involve 3-mer polymorphism types that have not yet been highlighted.

We next moved to our broadest, 7-mer sequence context model. Out of 20,665 possible 7-mer polymorphism types with sufficient data available for a statistical test, 142 surpassed Bonferroni multiple test correction (see Materials and Methods, and supplementary table 2*C*, Supplementary Material online). Of these, 117 represent expansions of the 3-mer signatures #1 and #2, whereas 25 have not been previously noted. The most prominent effect at the 7-mer level was the CAAACCC→C polymorphism (homogeneity test *P*_ordered_ = 3 × 10^−39^) corresponding to one of the Japanese-enriched 7-mers we identified above (table 2, fig. 3*A*). We also found that this polymorphism was variable across populations in the SGDP data set (*P*_ordered_ = 0.014), and that the pattern of relative enrichment and depletion in SGDP somewhat resembles that in 1KG (supplementary fig. 12, Supplementary Material online).

**Fig. 4.**
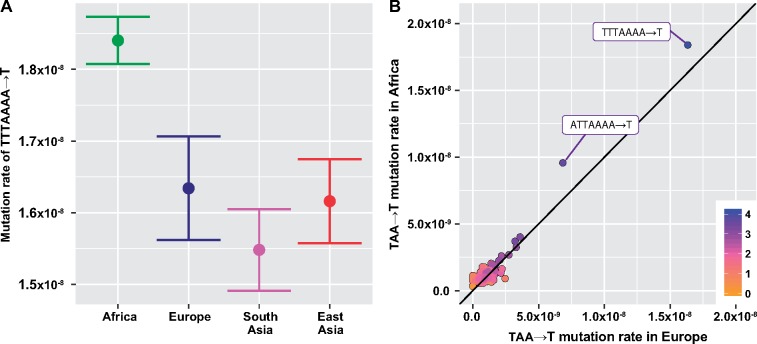
Two significantly variable 7-mer expansions of TAA→T. (*A*) Estimated private mutation rate of TTTAAAA→T across continental groups in 1KG, with approximate 95% confidence intervals shown. (*B*) TTTAAAA→T and ATTAAAA→T appear to be both more variable between continental groups and more common than other 7-mer expansions of TAA→T. These two polymorphism types are the only ones from the 256 TAA→T expansions that are significantly different (false discovery rate < 0.05) between Africa and Europe in 1KG.

Although we observed some 7-mer polymorphisms which were nominally significantly variable across populations in SGDP, in general we found that the SGDP set was simply too sparse to provide corroborating support (supplementary table 5 and supplementary fig. 13, Supplementary Material online). For those polymorphism types that did appear to harbor enough data to attempt replication, the relative patterns of enrichment and depletion across continents were mostly not consistent. One exception was TTTAAAA→T, which was heterogeneous in 1KG (*P*_ordered_ < 2 × 10^−21^), nominally significantly heterogenous in SGDP (*P*_ordered_ < 0.005), and showed an enrichment in Africa in both data sets, (fig. 4, supplementary fig. 14, Supplementary Material online). This corresponds to the 3-mer TAA→T, which is the 16th most significant polymorphism from our 3-mer-level heterogeneity analysis (*P*_ordered_ = 6.2 × 10^−36^). Examining the rates of the 7-mer expansions of TAA→T in 1KG, we find that TTTAAAA→T and ATTAAAA→T are outliers among other 7-mer expansions both in terms of their African enrichment and the overall number of polymorphisms of those types (fig. 4*B*), although the relative rates of ATTAAAA→T in 1KG and SGDP were not consistent (supplementary fig. 15, Supplementary Material online). This observation could indicate that the heterogeneity we observe in proportions of TAA→T polymorphisms is driven by an elevation of these two highly variable 7-mers in Africa, although the mechanism behind this is not clear. We note that a previous study identified the TTTAAAA motif as a hotspot for A-to-T mutations, with Alu/LTR transposase nicking as a potential mechanism (Carlson et al. 2018). We also note that several of 7-mer variants with sufficient counts of variants in both data sets for cross checking were repeat-rich (e.g., AAACAAA→A), which might indicate sequencing errors or other technical artifacts.

### Distribution of Derived Allele Frequencies within Mutational Signatures

**Fig. 5.**
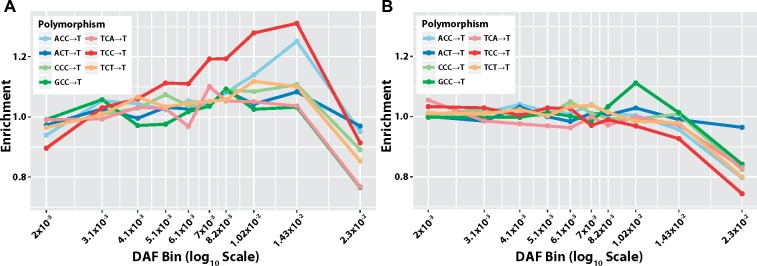
Enrichment of polymorphisms across DAF bins. The enrichment of polymorphisms in a signature across DAF bins in 1KG, calculated as the proportion of mutations in a bin with a given polymorphism class, divided by the proportion of all polymorphism with that class. (*A*) Signature #1 in Europeans. (*B*) Signature #1 in South Asians.

To better understand the mutational processes driving patterns of enrichment and depletion in certain polymorphism classes (fig. 1), we examined the distribution of derived allele frequencies (DAFs) of the polymorphisms in each signature. As in a recent publication (Harris and Pritchard 2017), we first separated polymorphisms into bins by DAF. For each bin, we then calculated the enrichment of each polymorphism type as its proportion in that DAF bin relative to its proportion across all DAF bins (see Materials and Methods). We highlight the DAF spectra of signature #1 in figure 5. The DAF spectra for more signatures in all continental groups are reported in the supplementary figures 16–19, Supplementary Material online. For signature #1, we observe the previously reported “pulse” of TCC→T mutations in Europe at ∼1% DAF (fig. 5*A*) and see the same pulse to a lesser degree in the additional signature #1 polymorphisms identified in this work. However, we did not observe this pulse for TCC→T polymorphisms among private variants in South Asia (fig. 5*B*), even though this continental group does have an overall enrichment of this signature. Only GCC→T, which in Europe has the least signature of the pulse, appears to be enriched around 1% DAF within South Asian private mutations. One explanation for the presence of signature #1 in South Asians is the result of admixture with Europeans, although further investigation is necessary to confirm or deny this hypothesis.

## Discussion

In this report, we describe several patterns of variability in polymorphism ratios between human populations at a global scale. Whether these patterns reflect a true difference in underlying mutational processes, and what those underlying causes might be, remains unclear. Even the most prominent signature, European C→T, is still poorly understood: Although this group of polymorphisms appears to correlate with mutational signatures linked to ultraviolet radiation or alkylating agents in one cancer study (Forbes et al. 2015; Mathieson et al. 2017), evidence supporting either of these causal mechanisms is limited (Mathieson et al. 2017). Moreover, although patterns of polymorphism enrichment across DAF spectra helped identify some groups of mutational changes which may have occurred in “pulses” in recent history, multiple polymorphism groups lacked an obvious pulse. This collection of observations may suggest further heterogeneity in the timing of specific events that may have contributed to these changes.

Although European C→T enrichment is by far the most prominent signature of variation (Harris and Pritchard 2017; Mathieson et al. 2017), the large number of variable polymorphism types and the variety of patterns they follow at a global scale suggest that several different processes are at work in shaping the ratios of polymorphisms we observe. The signatures we highlight here most-likely do not represent all distinct mutational processes that may have been actively operating in recent history. Although some of these signatures may be the result of sequencing artifacts, others may reflect true divergence in the underlying mutational process. In the latter case, further scrutiny of these differences may provide an opportunity to better understand the processes that shape genomic stability and genetic change. Moreover, quantifying and modeling polymorphism patterns as accurately as possible can help us fine-tune our predictions and interpretations of single-nucleotide genetic variation, potentially advancing our understanding of evolution or genetic disease.

One approach that may aid these efforts is the consideration of local genetic sequence context. Previous studies have identified patterns of heterogeneity in polymorphism levels that can be observed between polymorphisms from different 3-mer motifs of sequence context (Harris and Pritchard 2017), illustrating the importance of a single flanking base pair of context in shaping the probability of polymorphism. In this report, we consider a broader window of local sequence information, noting that although certain signatures are fully explained by a single flanking base pair or context, others appear to vary with sequence context up to 2–3 bp from the polymorphic site (figs. 2–4). We report that 10 of the 14 heterogeneous 7-mers between Chinese Dai and Japanese in signature #3 contain the 7-mer motif NNNACAG→C (table 2). In addition, we find that the apparent elevation of TAA→T 3-mer polymorphisms between Africa and Europe may in fact be driven by a strong enrichment of polymorphism within TTTAAAA contexts, which also appear to segregate more polymorphisms than other TAA contexts (fig. 4*A* and *B*).

There are some limitations to note in our study. First is sample size: Although broader sequence context models can capture more information, they can also require much more total genetic data to be sufficiently well-powered for our statistical analysis. This is made especially difficult because asking comprehensive questions about global mutation rate patterns requires a large and ethnically diverse data set of genetic variation, the likes of which are only recently becoming available. For example, in this report, we noted evidence suggesting that East Asian heterogeneity in NAC→C and TAT→T mutations may be strongest on the X chromosome (table 2). Given this observation, it may be informative to examine the dispersion of these polymorphisms across the X-chromosome, because a genetic variant responsible for an increase in mutation rate is likely to be found in a genetic context with high rates of polymorphism (Seoighe et al. 2017). Unfortunately, however, a problem of power quickly emerges, because the total number of polymorphisms of any 7-mer context observed on the X chromosome is still relatively small. As a result, analyses regarding this signature may be difficult until a larger amount of East Asian genetic data is made available. In addition, corroborating results in the SGDP data set may give us some idea of how consistent a signature is across data sets and rule out some biological mechanisms. However, this data set is itself not perfectly representative of the corresponding 1,000 Genomes populations, and it is far smaller, presenting limitations in terms of comparability and statistical power. Additional, deeply sequenced samples from diverse populations would be ideal for further targeted hypothesis testing, validation, and improvement of the mathematical models designed to capture this global mutation rate variability.

A second complication is that signals of polymorphism enrichment from population-level data may reflect some distant ancestral mutation rate variation, and some from more recent history. As such, it is not immediately clear whether the biological mechanisms driving these phenomena are still active today. Measurements of enrichment of these polymorphism types in ancient DNA and across allele frequency bins suggest that the previously reported European signal (signature #1) may correspond to an ancestral increase in the rate of certain C→T mutations ∼15,000 years ago, which may have subsided ∼2,000 years ago (Harris and Pritchard 2017; Mathieson et al. 2017). In this study, we examined the distribution of polymorphism enrichment across allele frequencies, allowing us to hypothesize about the timing of these events across continental groups (fig. 5). Further analyses might infer the timing of polymorphism from statistical model-based estimates of allele age or from ancient DNA as in Mathieson et al. (2017). This may help us better piece together the timescale over which mutation rates may have changed.

It is likely that further investigation will reveal details of mechanism, evolutionary timing, and genome-wide or subpopulation-level patterns in polymorphism variation, and our report here is by no means exhaustive. We detail evidence suggesting that polymorphism variation acts in a variety of ways across human populations based on local sequence context cues at varying distances from the mutated locus. Although some of these signals manifest at the 3-mer level, consideration of a broader context brings new patterns of variation to light.

## Materials and Methods

### Compilation of Private Variant Sets

Variants from the 1000 Genomes Project release (downloaded February 26, 2016, phase 3) (Auton et al. 2015) were filtered to include single nucleotide polymorphisms, excluding multiallelic variants, in/dels, and any variants with a filter tag other than “PASS.” Based on the exclusion criteria from previous work, we also omitted variants in coding regions, centromeres, telomeres, and additional sections of the genome predicted to have low accessibility (Aggarwala and Voight 2016). From this set, we considered variants with a minor allele count 2 or greater across all samples. Although including singleton variants (those observed only once in the data set) in theory would provide more information about the recent de novo mutation rate, previous efforts to analyze human polymorphism variation with singletons have proven difficult to replicate (Harris 2015), so we opted to exclude them from our analyses.

From variants that passed our filters, we compiled lists of variants “private” to each nonadmixed continental group from the data set: Africans from Africa (AFR), Europeans (EUR), East Asians (EAS), and South Asians (SAS). We defined a single nucleotide polymorphism as “private” to a continental group if it is observed in that group but not in each of the other three. For all analyses, Americans of African Ancestry in Southwest USA (ASW) and African Carribeans from Barbados (ACB) were considered to be admixed American populations and were omitted from the African continental group.

For subpopulation-level analyses, we passed along private polymorphisms defined for each continental group into each respective subpopulation list. For example, a polymorphism which was private to AFR and observed in both Kenya and Gambia would be added to the subpopulation lists for both LWK (Luhya in Webuye, Kenya) and GWD (Gambians in Western Divisions in Gambia). All filtration steps were carried out using vcftools and the vcf-isec tool (v0.1.12b) (Danecek et al. 2011).

From each continental or subpopulation list, we tallied counts of private variants by increasingly broader windows of sequence context, including one, two, and three flanking nucleotides (i.e., 3-mer, 5-mer, and 7-mer context, respectively). During this process, mutation classes were “folded” to include its reverse-complimentary equivalent (e.g., TCC→T and GGA→A were considered together). Code utilized for each step is available online (github.com/raikens1/mutation_rate; last accessed February 26, 2019).

To obtain the private variant lists in SGDP, the same filtration criteria were used as for the 1KG data, except that sites with >20% missing data in the population were discarded.

### Statistical Comparison with Homogeneity Test

To replicate previous work by Harris and Pritchard (Harris 2015; Harris and Pritchard 2017), we first performed pairwise chi-square comparisons of polymorphism count between each possible pair of populations for each 3-mer polymorphism type (supplementary fig. 1, Supplementary Material online). Next, to partially relieve the multiple testing burden of six pairwise population comparisons over each possible mutation type, we performed a single test using a 2 × 4 cell contingency table which included polymorphism counts for each continental group. The resulting chi-square test for homogeneity from this table has three degrees of freedom. An issue with performing this type of analysis across all polymorphism types is that the *P*-values from these tests are nonindependent: A polymorphism that is profoundly heterogeneous across populations may alter the proportions of other polymorphism types. For these reasons, we used the *P*_ordered_ correction as previously described (Harris and Pritchard 2017). Using this procedure, each polymorphism type is initially tested and ranked according to increasing significance based on a simple homogeneity test using all the data. A corrected *P*-value is then calculated for each polymorphism. To do this, the least significant polymorphism is assigned its original *P*-value using all of the data. After this, the *P*-value for the *i*th least significant polymorphism type is recalculated using a homogeneity test with only the data for the *i* least significantly variable polymorphisms from the initial ranking. All chi-square comparisons were performed using the chisq.test function in R (v3.4.0), and significance thresholds were determined based on a Bonferroni correction with a nominal error rate (α = 0.05).

### Mutation Rate Inference

The probability of observing a given polymorphism in a population is determined by a composite of mutation rate, demography, sample size, and other factors (Watterson 1975). To facilitate comparisons across populations, we calculated a mutation rate, calibrated to the average de novo mutation rate estimated by Kong et al, which assumes a paternal average age of 29.7 (Kong et al. 2012). Assuming all populations have a total mutation rate of 1.2 × 10^−8^, we inferred the mutation rate of a specific type (say TCC→T) as

(1) $$\mu_{m}=\;1.2\times 10^{-8}\times \Theta^{-1}\times \theta_{m},$$

where μ_m_ represents the inferred private germline TCC→T mutation rate per generation per site, θ_m_ represents the proportion of all TCC sites in the genome with private C/T polymorphism in the population, and Θ represents the total proportion of all sites of any type in the genome which are private polymorphisms in the population. Under this formulation, Θ can be written as a weighted sum of the θ_m_ over all mutation types, where the weight for θ_m_ is given by the proportion of sites in the genome which have the sequence context for mutation type *m*. This gives a genome wide mutation rate of 1.2 × 10^−8^; 95% confidence intervals for μ_m_ were calculated using the normal approximation to the binomial, assuming the variance in our estimate of Θ^−1^ to be approximately zero.

### Clustering Polymorphism Types

We used the heatmaps 2 hierarchical clustering methods from the basic stats package in R (v3.4.0) in order to heuristically identify mutation types that vary in similar ways across the globe. In doing so, we defined the “signature” of a mutation *m* across a set of populations as a vector of the inferred mutation rate of *m* in each population. Each pattern of rates across populations was normalized by fold difference above or below the mean rate for that signature. We used Euclidean distance to construct each heatmap, selected because these methods gave the most clearly interpretable results and agreed the most closely with previous work (Harris 2015; Harris and Pritchard 2017; Mathieson et al. 2017)

### Testing for Enrichment of Signature #3 on the X Chromosome

In order to test for enrichment on the X chromosome (table 2), we used a one-sided binomial test to determine whether the observed proportion of privately polymorphic sites of a given substitution type on the X chromosome was greater than *p*_0_, the expected proportion under the null hypothesis. For demographic and sampling reasons, we expect to observe fewer polymorphic sites of any given type on the X chromosome than on the autosomes, even if the mutation rate of that polymorphism type is identical across chromosomes. Thus, to estimate *p*_0_, we first needed to calculate the ratio, *ξ*, of X-chromosome polymorphism probability to autosomal polymorphism probability across all other substitution types. Estimating *ξ* as the ratio of the proportion of sites polymorphic on X versus the proportion of sites polymorphic on the autosomes, we then used this as a scaling factor, estimating *p*_0_ as *ξp*_A_, where *p*_A_ represents the maximum likelihood estimate for the polymorphism probability for that polymorphism across all autosomes. This gives the null hypothesis estimate *p*_0_ for the probability of polymorphism on the X chromosome for the context of interest, assuming that the mutation rate on X and the autosomes is the same.

### Enrichment across DAF Bins

To identify the DAF of mutations private to each continental group, we used the ancestral allele calls reported in the 1000 Genomes Phase 3 release VCF files. These ancestral calls are originally from the Ensembl Compara release 59 (Zerbino et al. 2018). For the purposes of this specific analysis, we omitted variants that did not have a high-confidence ancestral state call, and variants on the X chromosome. For each continental group, we binned variants by identifying the quantiles of DAFs by 5% increments and collapsing to only quantiles with unique values (because DAF is not continuous in finite populations). In this way we tried to ensure that each bin had a reasonable amount of data, though not all bins were the same size. We calculated enrichment of polymorphisms within a sequence context in each bin as the proportion of polymorphisms in that bin with that context, divided by the proportion of all polymorphisms with the given context across all bins.

## Supplementary Material

Supplementary Data
