# Supplementary material for: Signals of Variation in Human Mutation Rate at Multiple Levels of Sequence Context
Source: Mol Biol Evol. 2019 Feb 7;36(5):955–65. doi: 10.1093/molbev/msz023 (PMC6501879; doi:10.1093/molbev/msz023)
Supplement: Supplementary Data [file msz023_supp.zip › supplement_r03.pdf]

Supplementary Notes

Rachael C. Aikens, Kelsey E. Johnson, Benjamin F. Voight

July 6, 2018

Supplementary Tables

A.

Study	Africans	Europeans	East.Asians	South.Asians
1,000 Genomes	504	503	504	489
SGDP	44	69	47	39

B.

Study	Africans	Europeans	East.Asians	South.Asians
1,000 Genomes	7.0	1.3	2.0	2.0
SGDP	3.6	0.7	0.5	0.3

Supplementary Table 1: Sample sizes in terms of (A) individuals and (B) polymorphic sites (in millions) for each ancestral population in 1,000 genomes and SGDP.

Supplementary Table 2: *Available for download in csv format.* All (A) 3-mer, (B) 5-mer, and (C) 7-mer subsitutions significantly heterogeneous across ancestral groups in 1,000 genomes after ordered p-value correction. Relevant sub-contexts, counts in each population, and total number of contexts in the included regions of the genome are shown for each significant substitution type. Substitutions are listed in decreasing order of significance.

Supplementary Table 3: *Available for download in csv format.* All 3-mer substitutions significantly heterogeneous across ancestral groups in SGDP after ordered p-value correction. Counts in each population and total number of contexts in the included regions of the genome are shown for each significant substitution type. Substitutions are listed in decreasing order of significance.

Supplementary Table 4: *Available for download in csv format.* All 3-mer substitutions significantly heterogeneous across ancestral groups in both SGDP and 1,000 genomes ($p < 5 \times 10^{-4}$) after ordered p-value correction. There are many reasons to expect that the exact numeric estimates of private mutation rate do not agree between SGDP and 1,000 Genomes, such as sequencing artifacts or differences in the populations sampled by the two data sets. As a result, "agreement" between the datasets was determined based on the relative enrichment and depletion of polymorphisms across continents, rather than the absolute mutation rate estimates. Substitutions are listed in decreasing order of significance in 1,000 genomes.

Supplementary Table 5: *Available for download in csv format.* All 24,576 possible 7-mer substitutions in both SGDP with annotation for whether hypothesis testing was considered feasible. A hypothesis test was considered impractical if there were fewer than 5 observed substitutions observed in one or more cells of the chi squared table. Relevant sub-contexts, counts in each population from SGDP, and total number of contexts in the included regions of the genome are shown for each significant substitution type.

Supplementary Figures

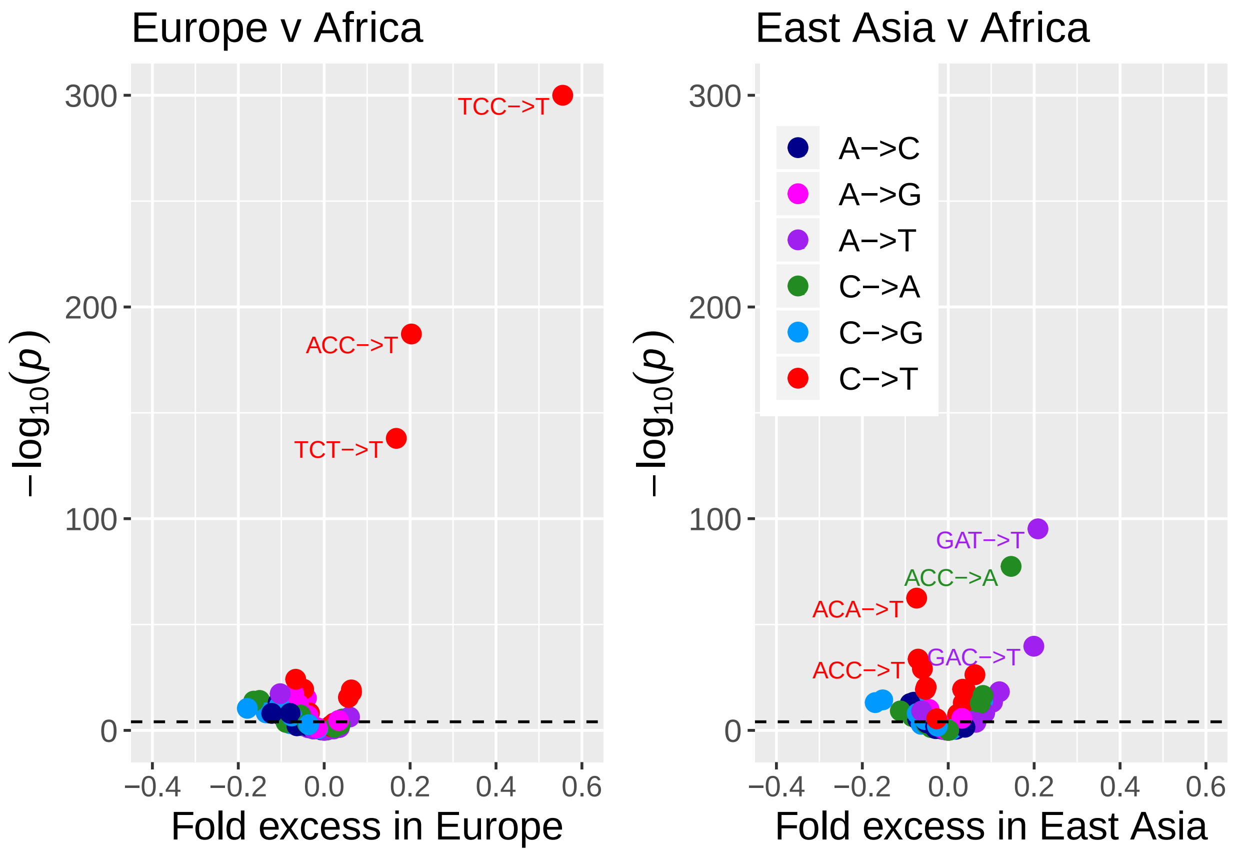

Supplementary Figure 1: *Replication of Figure 1 from Kelley Harris, 2015.* Using our variant filtration pipleline for extracting population-private variants from the Phase III 1,000 Genomes Dataset, we recapitulate Harris's previous findings from analysis of the Phase I Release. In keeping with Harris, 2015, the proportions of private 3mer substitutions were compared between pairs of populations using a pairwise chi-squared test. Ordered p-value correction was not applied. Unlike Harris, we choose to consider reverse-complimentary substitution classes as identical (e.g. TCC→TTC and GGA→ GAA are considered equivalent). The p-value for TCC→T in Europe versus Africa was too small to be represented in R; here it is shown rounded to $1{\times}10^{-300}$.

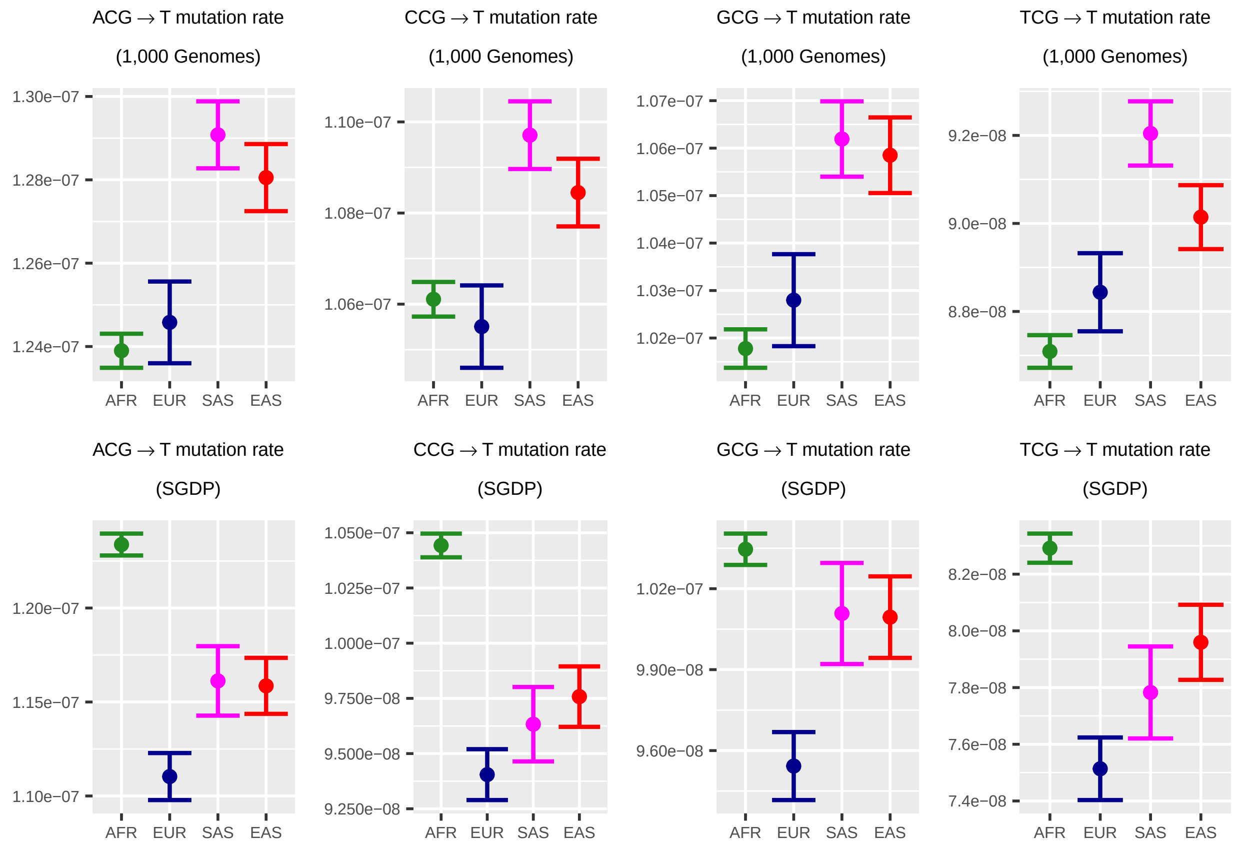

Supplementary Figure 2 Approximate 95% confidence interval estimates of inferred private mutation rate for each 3-mer CpG transisition across Africa (AFR), Europe (EUR), South Asia (SAS), and East Asia (EAS). Estimates shown are from 1,000 Genomes (A-D) and SGDP (E-H).

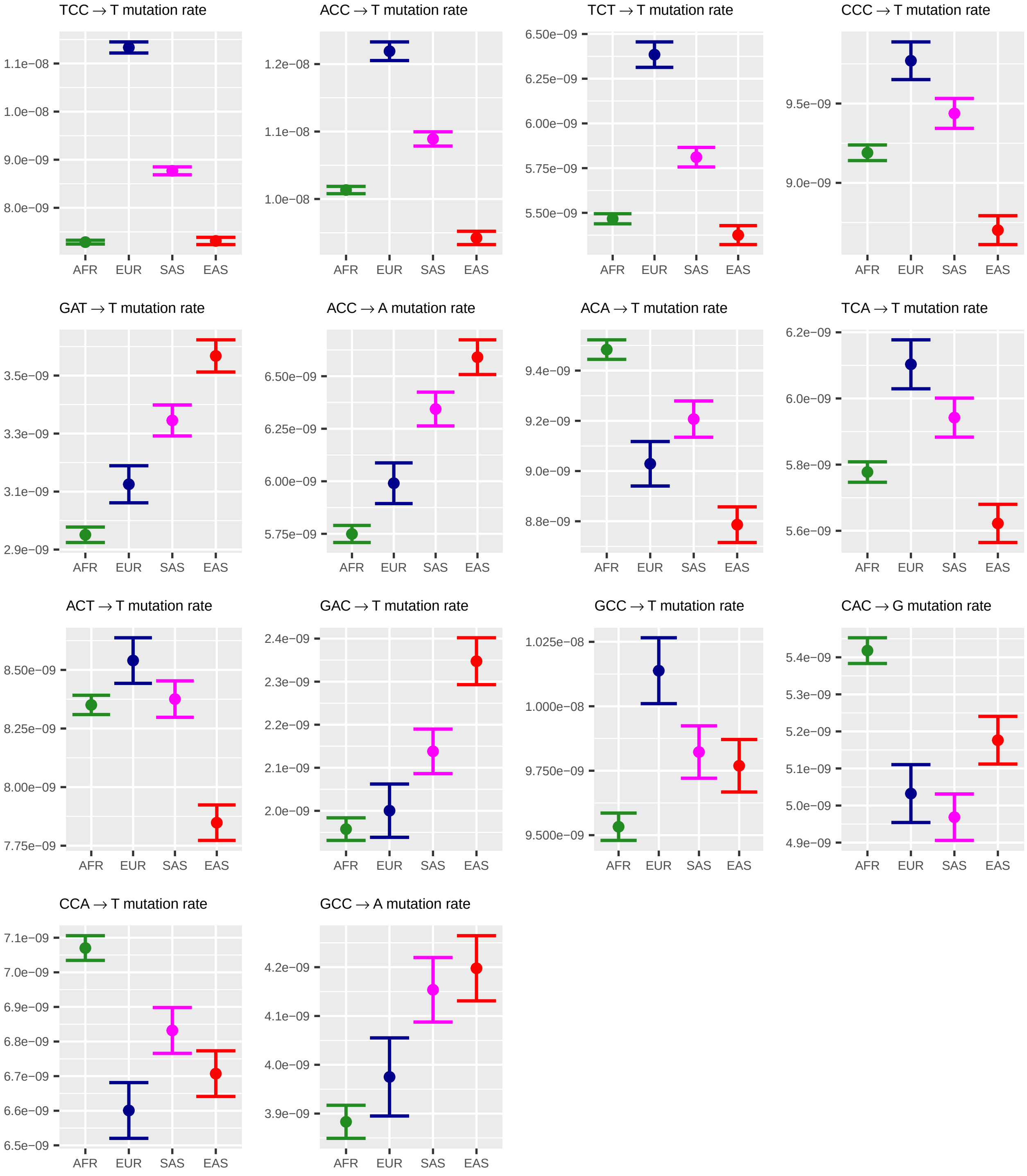

Supplementary Figure 3: Approximate 95% confidence interval estimates of inferred mutation rate in 1,000 Genomes for each variable 3-mer type listed in Table 1 of the main text. Rates are shown across Africa (AFR), Europe (EUR), South Asia (SAS), and East Asia (EAS).

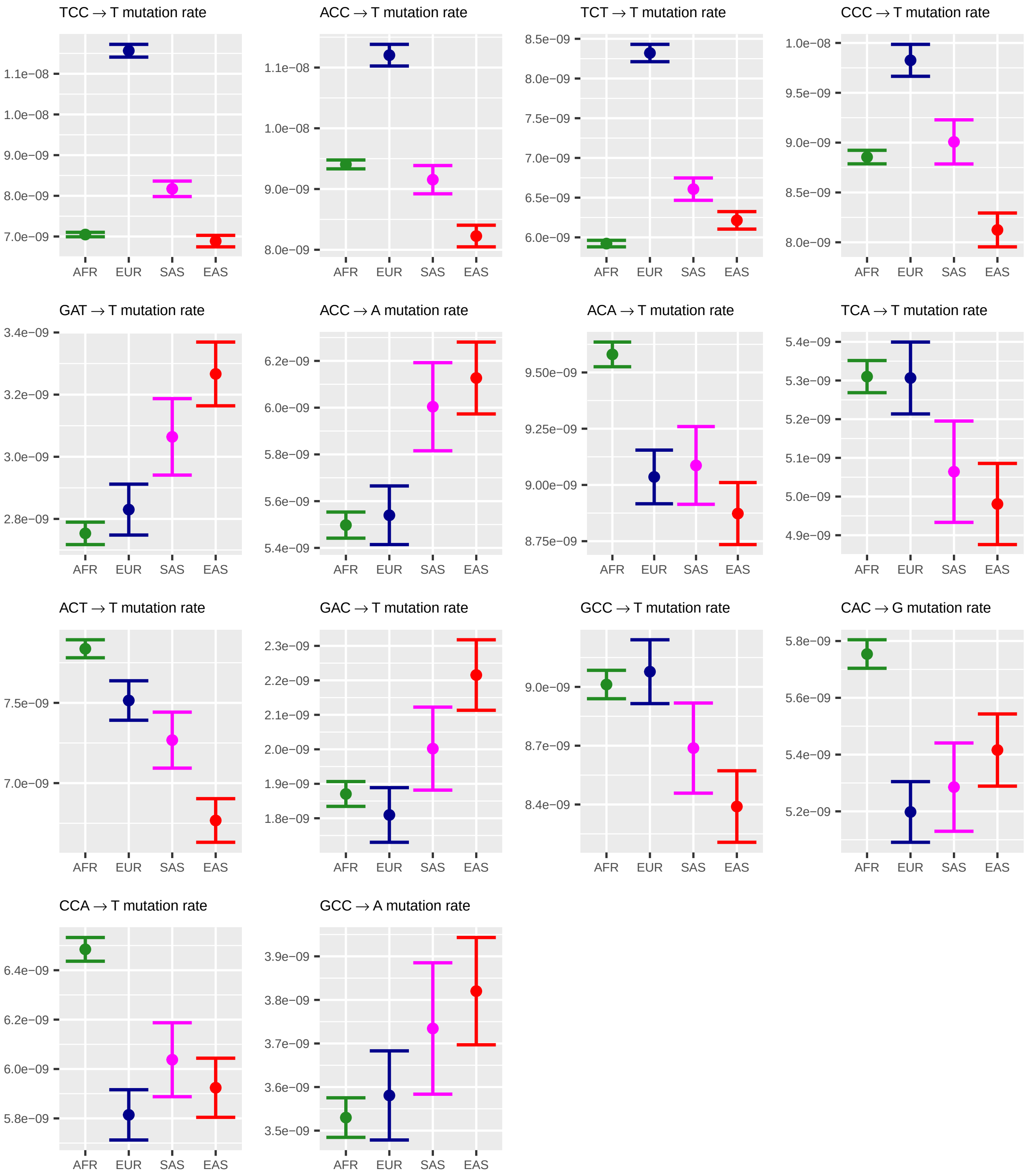

Supplementary Figure 4: Approximate 95% confidence interval estimates of inferred mutation rate in SGDP for each variable 3-mer type listed in Table 1 of the main text. Rates are shown across Africa (AFR), Europe (EUR), South Asia (SAS), and East Asia (EAS).

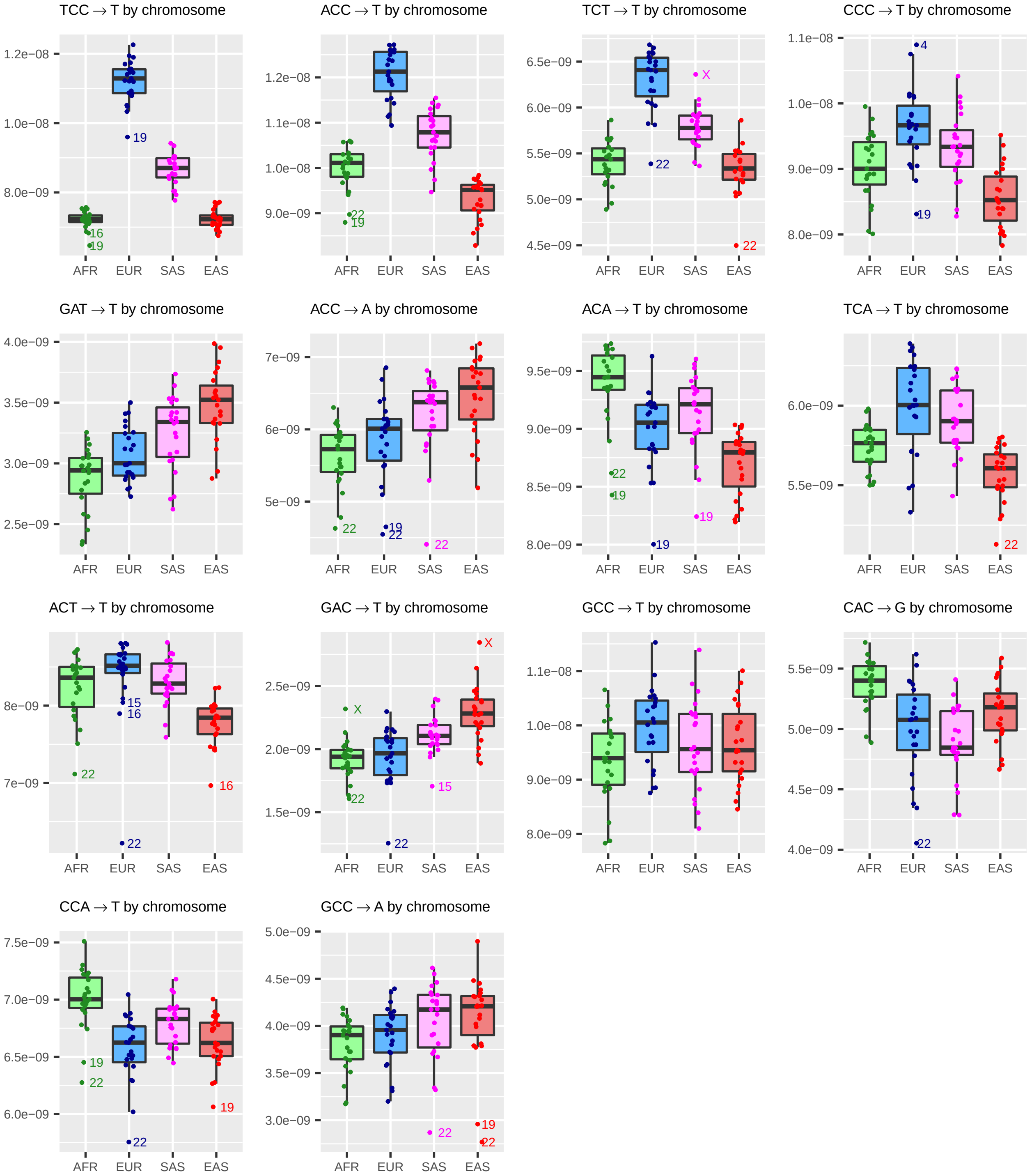

Supplementary Figure 5: Box plots of inferred private mutation rate across chromosomes in 1,000 Genomes for each variable 3-mer type listed in Table 1 of the main text. Outlier points are labeled with chromosome.

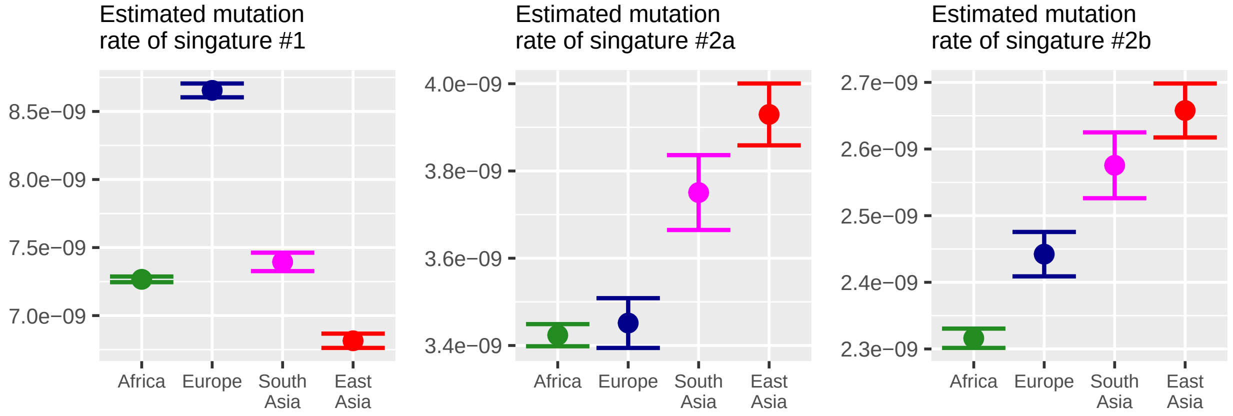

Supplementary Figure 6: Approximate 95% confidence intervals for estimated pooled mutation rate in SGDP for (A) signature #1, (B) signature #2a, and (C) signature #2b.

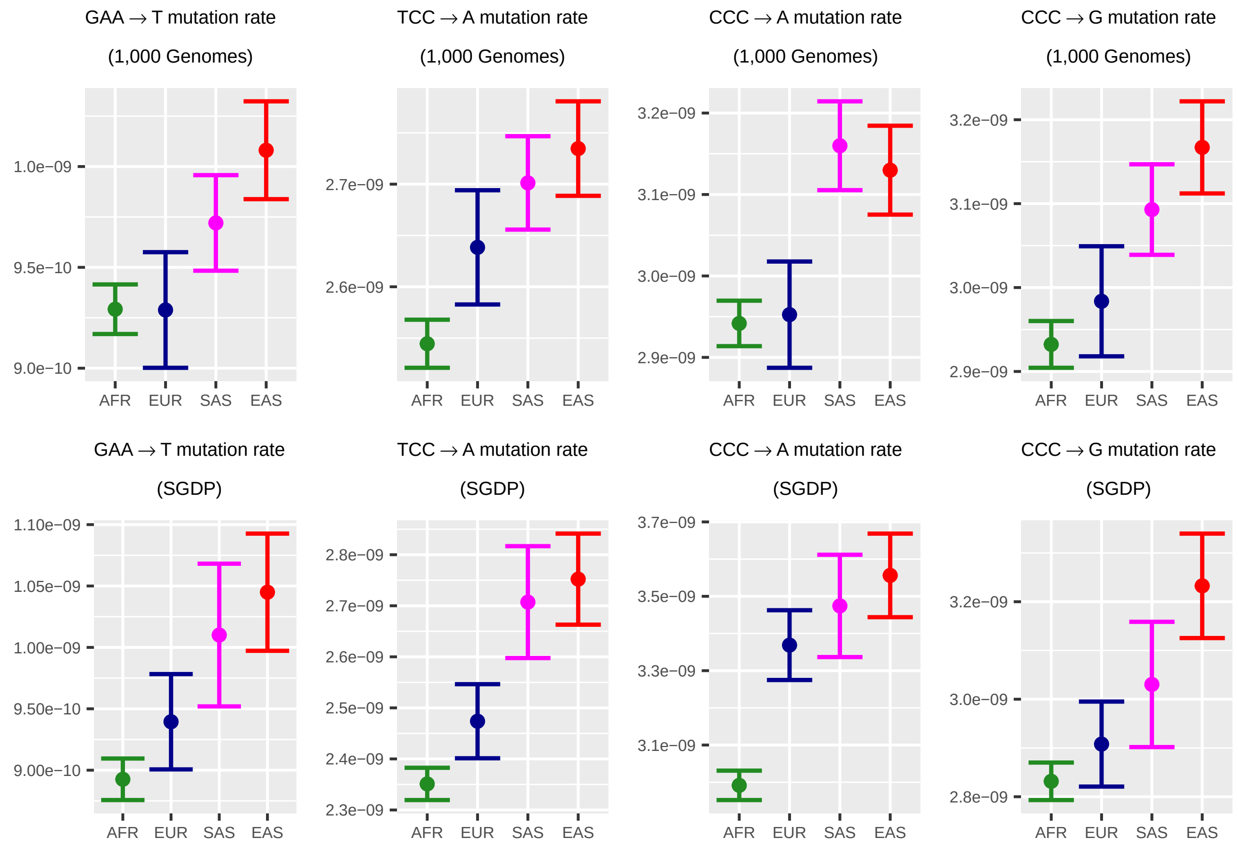

Supplementary Figure 7: Approximate 95% confidence intervals of estimated mutation rate for all signature #2 polymorphisms not already shown in Supplementary Figure 3 and Table 1. Rate estimates are shown for Africa (AFR), Europe (EUR), South Asia (SAS), and East Asia (EAS) within 1,000 Genomes (A-D) and SGDP (E-H).

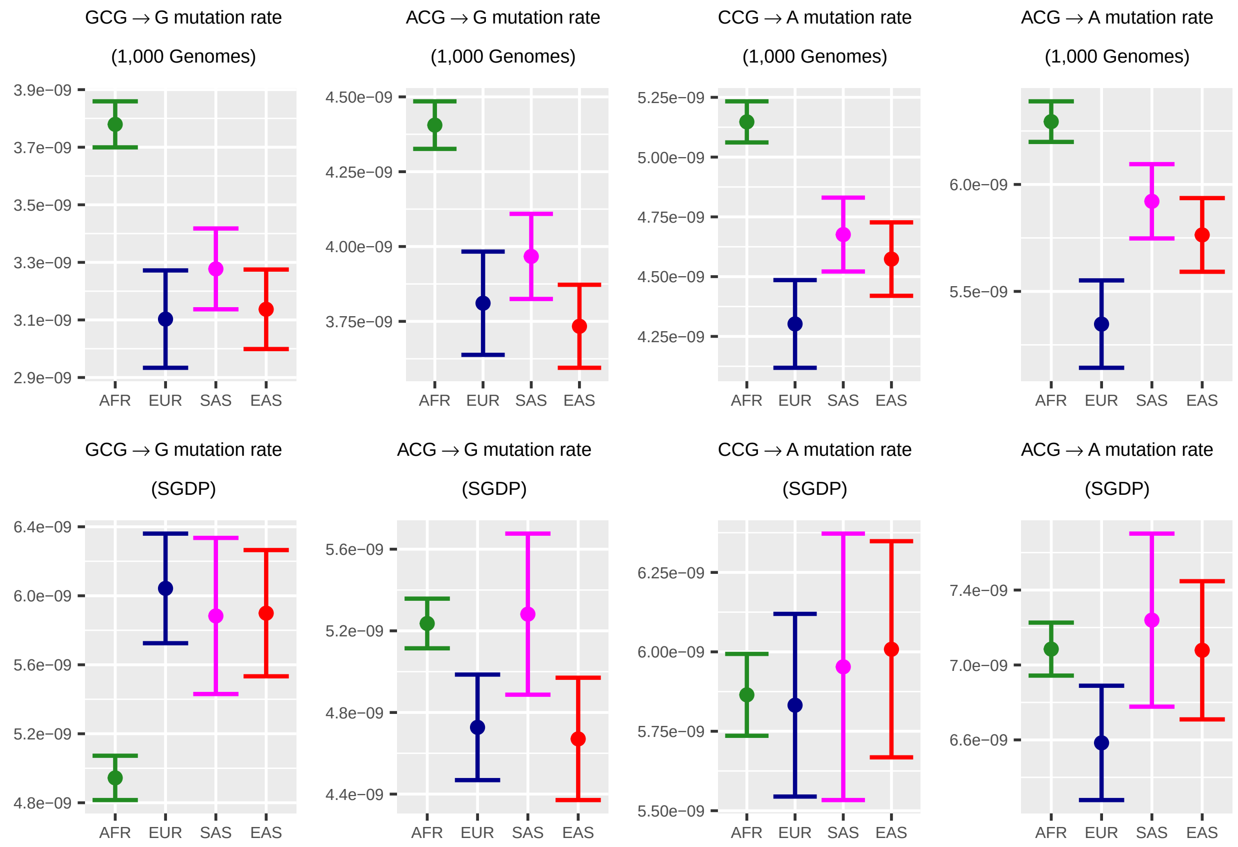

Supplementary Figure 8: Approximate 95% confidence intervals of estimated mutation rate for all signature #5 polymorphisms. Rate estimates are shown for Africa (AFR), Europe (EUR), South Asia (SAS), and East Asia (EAS) within 1,000 Genomes (A-D) and SGDP (E-H).

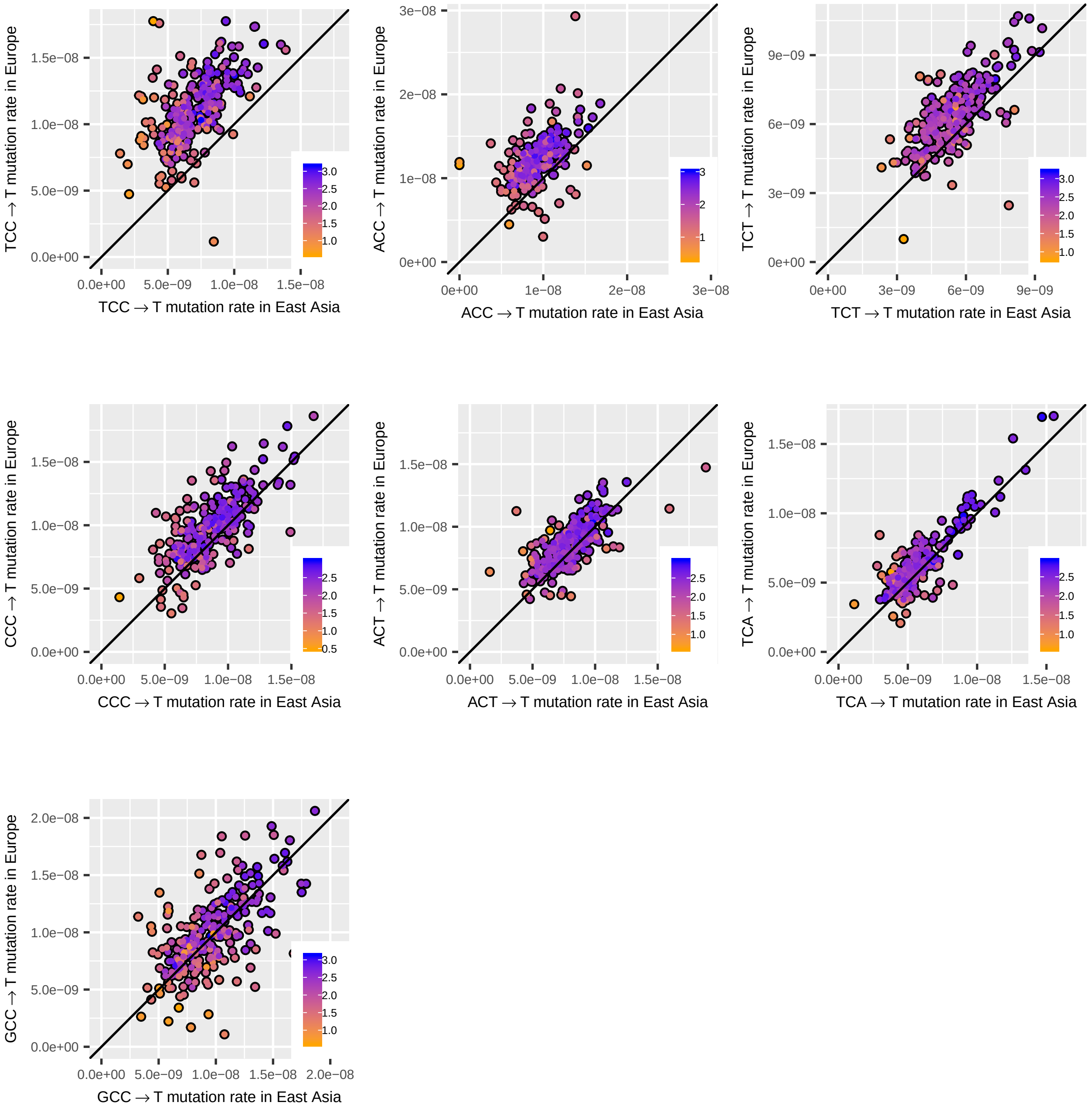

Supplementary Figure 9: Rates of all 7-mer expansions of each signature #1 polymorphism in Europe versus East Asia. Each point represents a 7-mer expansion of the 3-mer subtype shown, plotted based on its estimated mutation rate in each of the two populations displayed. Colors indicate the log (base 10) of the number of substitutions observed for that 7-mer class. Europe and East Asia were selected to visualize this comparison because the difference in mutation rate for signature #1 3-mers is most strong and consistent between these populations. Generally, these polymorphisms match case II (see main text): enrichment is consistent across 7mers, with some expected amount of noise.

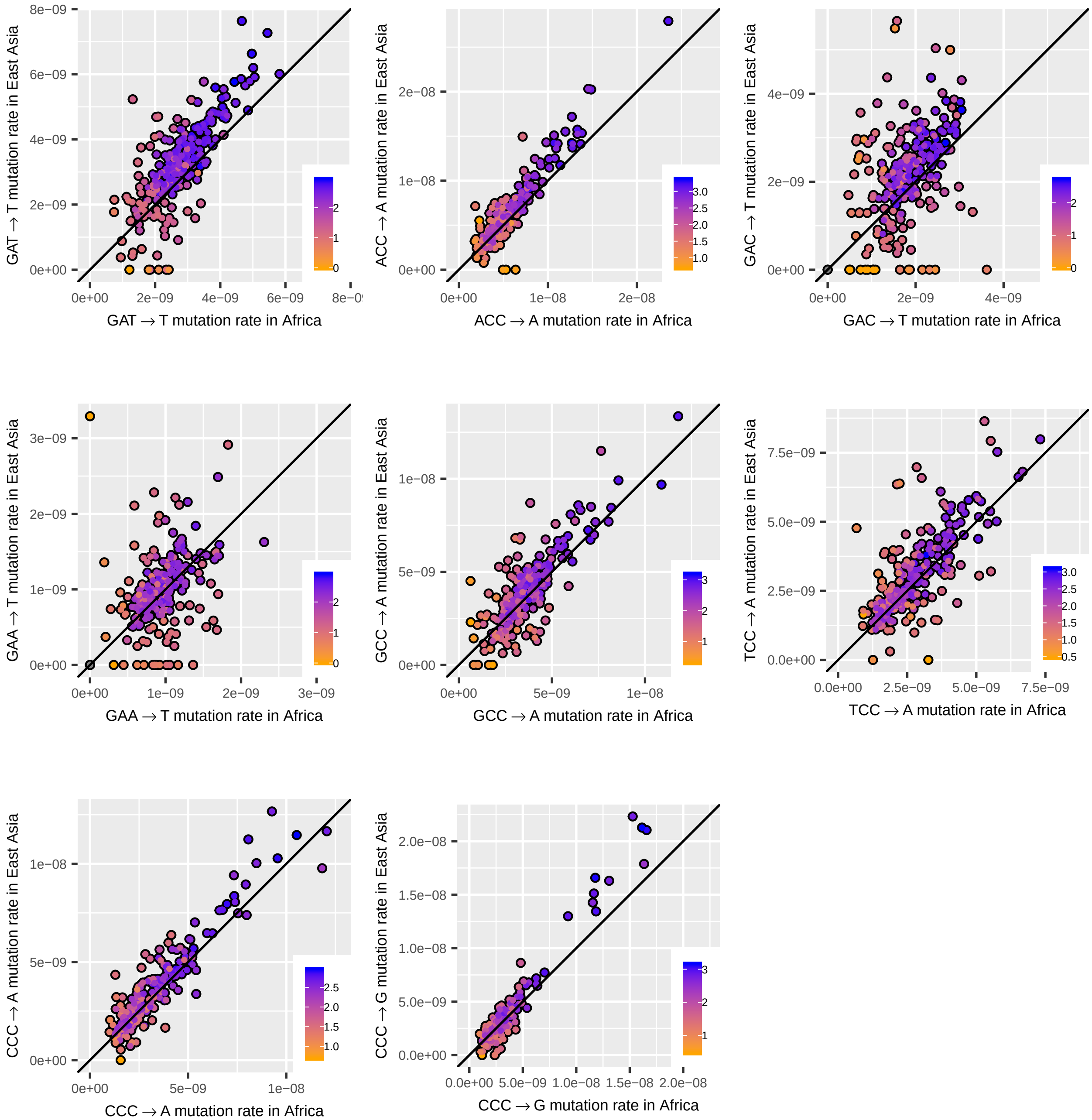

Supplementary Figure 10: Rates of all 7-mer expansions of each signature #2 polymorphism in Africa versus East Asia. Again, the population-specific enrichment is consistent across 7mers, consistent with case II.

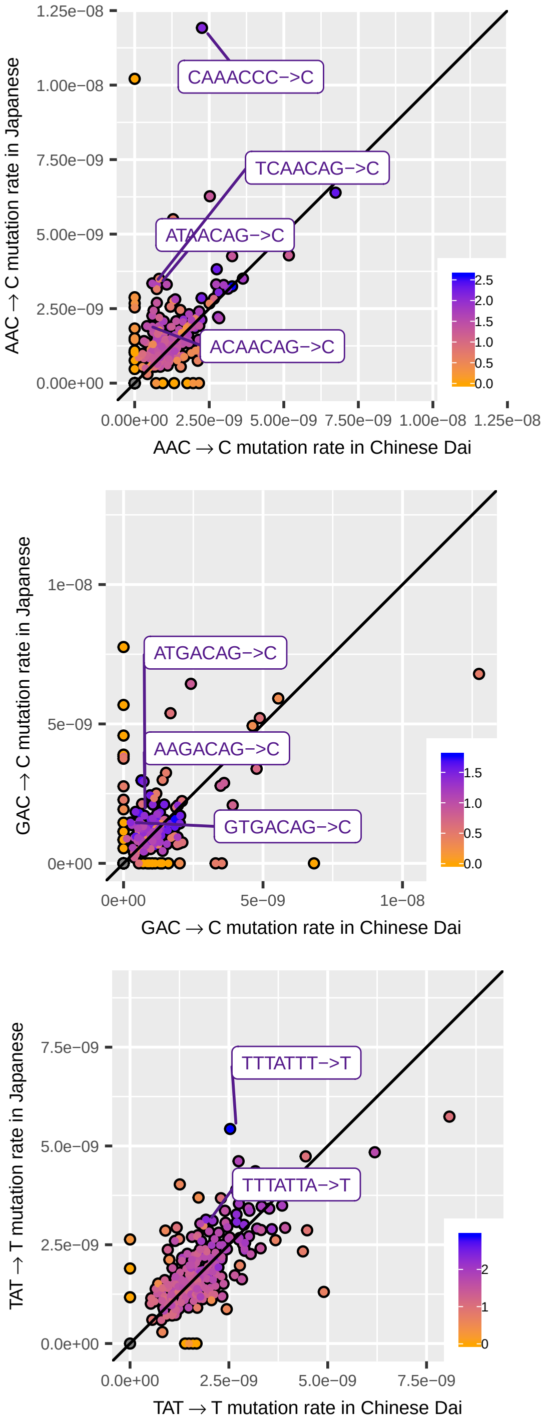

Supplementary Figure 11: Rates of all 7-mer expansions of each signature #3 signature in Japanese versus Chinese Dai. Labeled points are those which were significantly variable between Japanese and Chinese Dai (fdr < 0.05 among all signature #3 7-mer expansions).

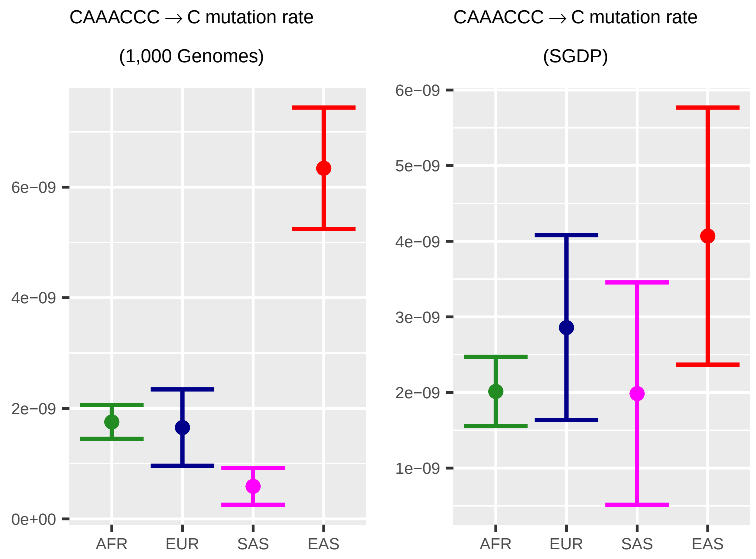

Supplementary Figure 12: Approximate 95% confidence intervals of estimated mutation rate for CAAACCC→C in Africa (AFR), Europe (EUR), South Asia (SAS), and East Asia (EAS). Estimates were made from (A) 1,000 Genomes, and (B) SGDP. Note: Only seven CAAACCC→C variants were observed in South Asia withn SGDP, so estimation of this quantity is highly uncertain.

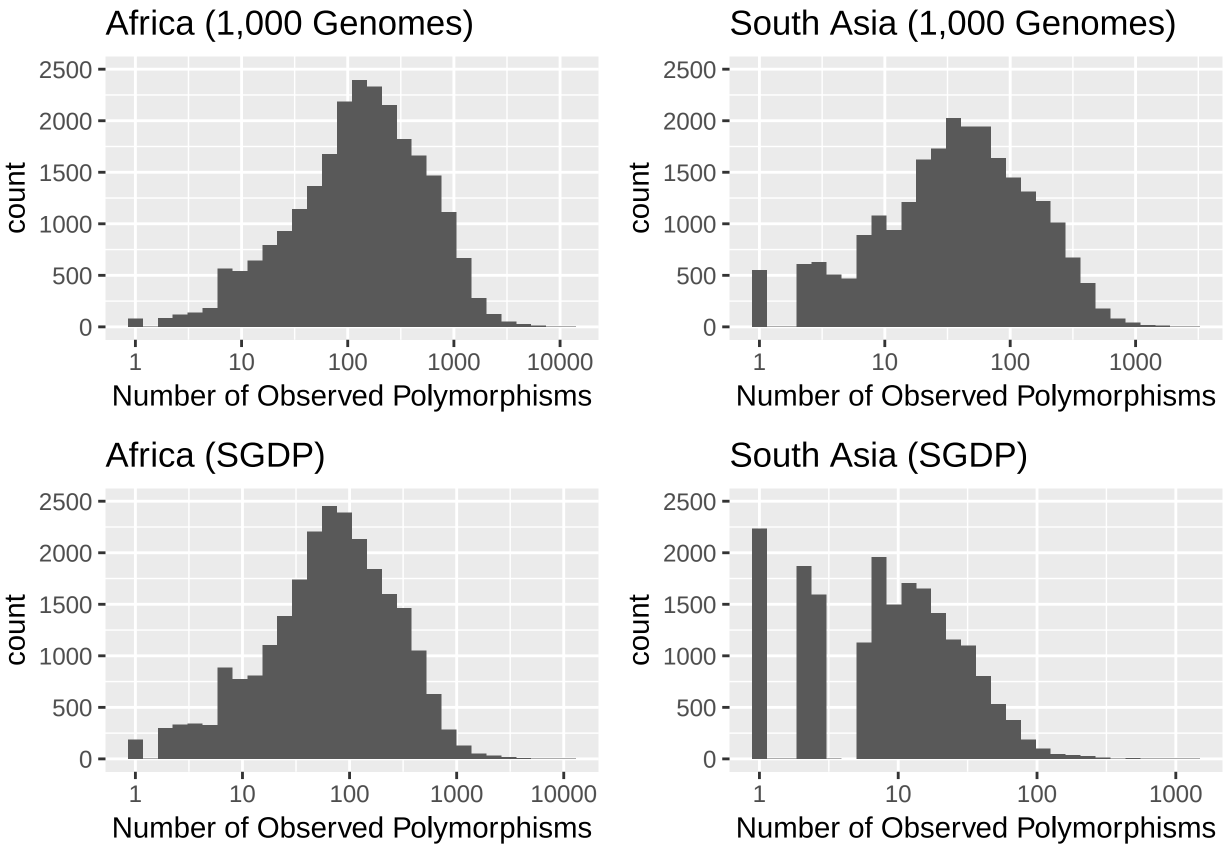

Supplementary Figure 13: Histogram of the number of observed polymorphisms for all 24,576 possible 7mers in representative populations from 1,000 Genomes and SGDP. The populations shown are Africa and South Asia because these are the populations with the most and the fewest observed polymorphisms overall (respectively). All x-axes use a log base 10 scale.

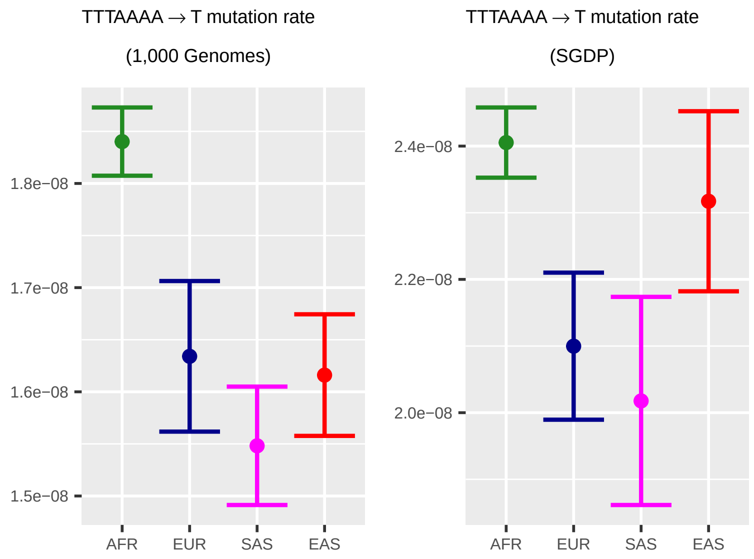

Supplementary Figure 14: Approximate 95% confidence intervals of estimated mutation rate for TTTAAAA→T in Africa (AFR), Europe (EUR), South Asia (SAS), and East Asia (EAS). Estimates were made from (A) 1,000 Genomes, and (B) SGDP.

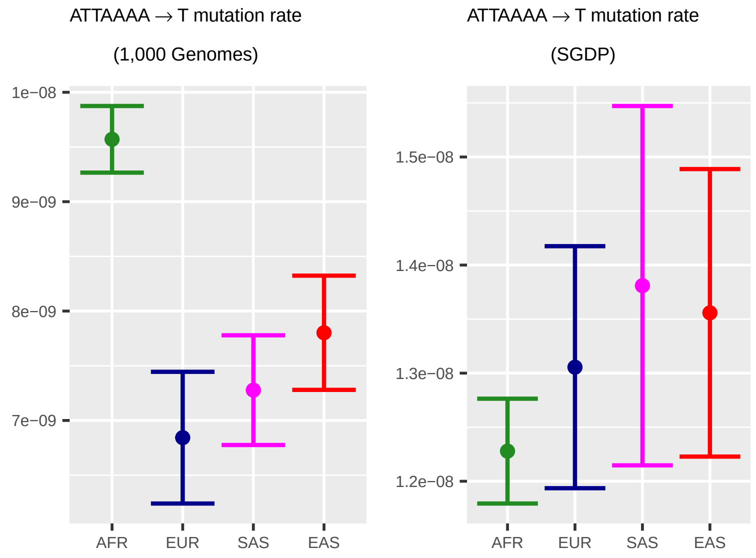

Supplementary Figure 15: Approximate 95% confidence intervals of estimated mutation rate for ATTAAAA→A in Africa (AFR), Europe (EUR), South Asia (SAS), and East Asia (EAS). Estimates were made from (A) 1,000 Genomes, and (B) SGDP.

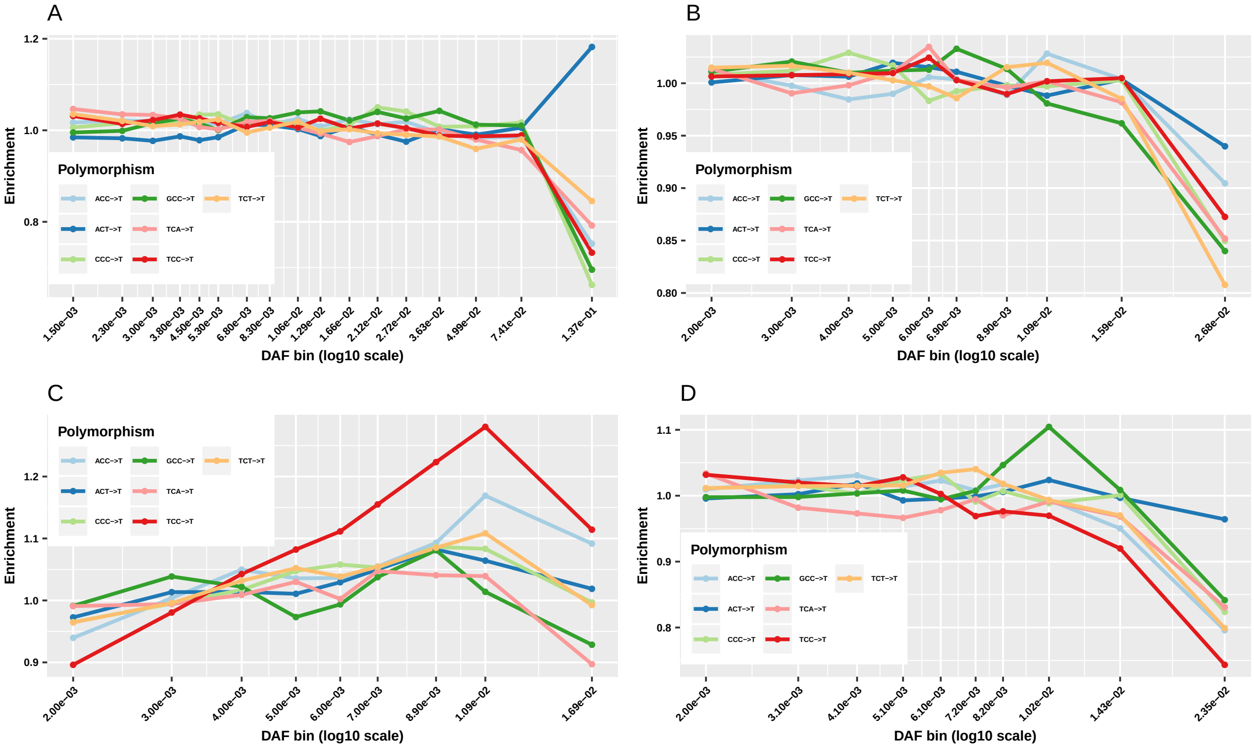

Supplementary Figure 16: DAF enrichment for singature #1 in (A) Africa, (B) East Asia, (C) Europe, (D) South Asia.

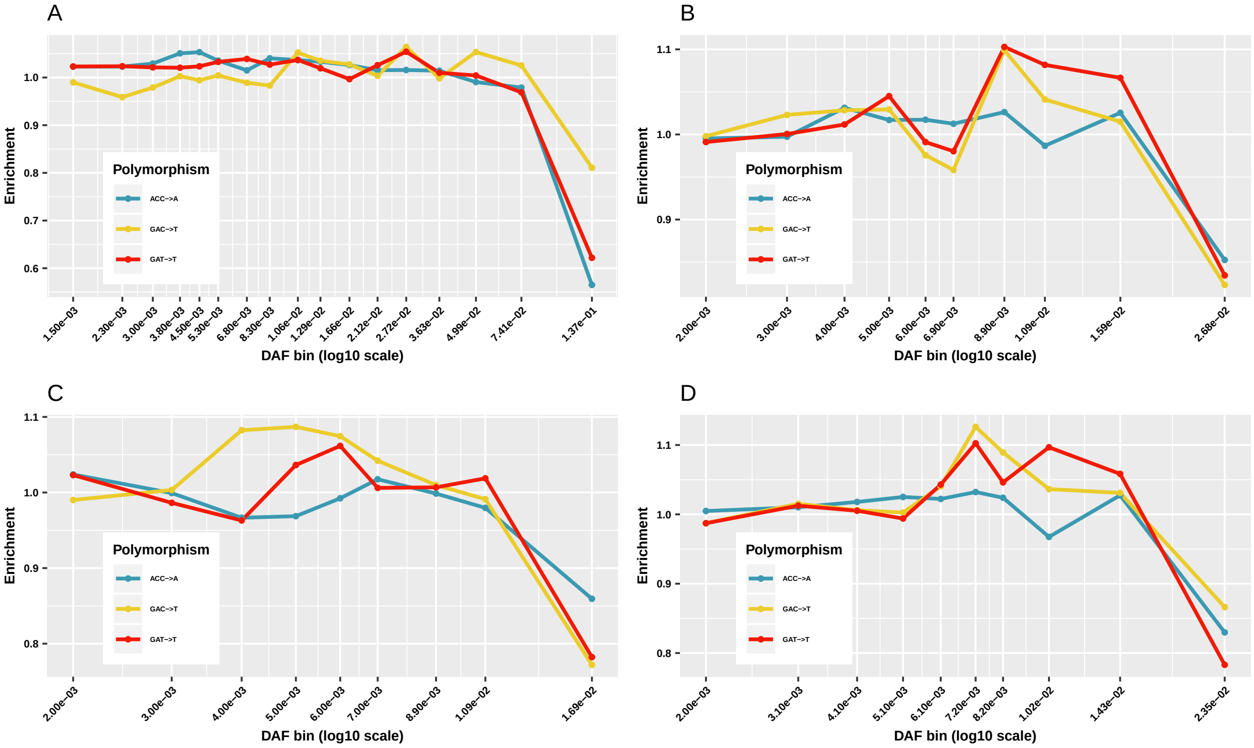

Supplementary Figure 17: DAF enrichment for singature #2a in (A) Africa, (B) East Asia, (C) Europe, (D) South Asia.

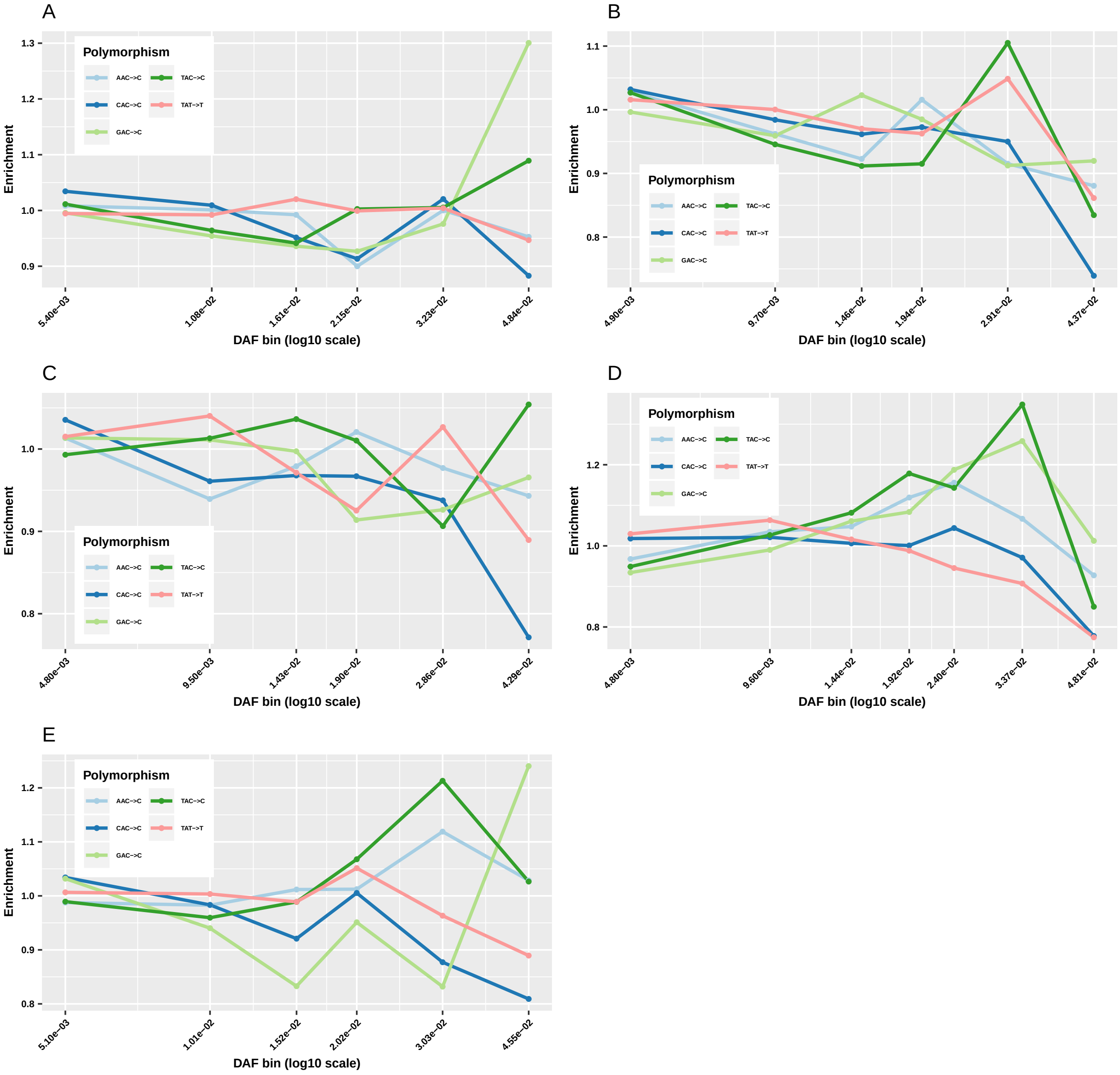

Supplementary Figure 18: DAF enrichment for 3-mers in singature #3 in (A) Chinese Dai, (B) Han Chinese from Beijing, (C) Han Chinese from Southern China, (D) Japanese, (E) Vietnamese.

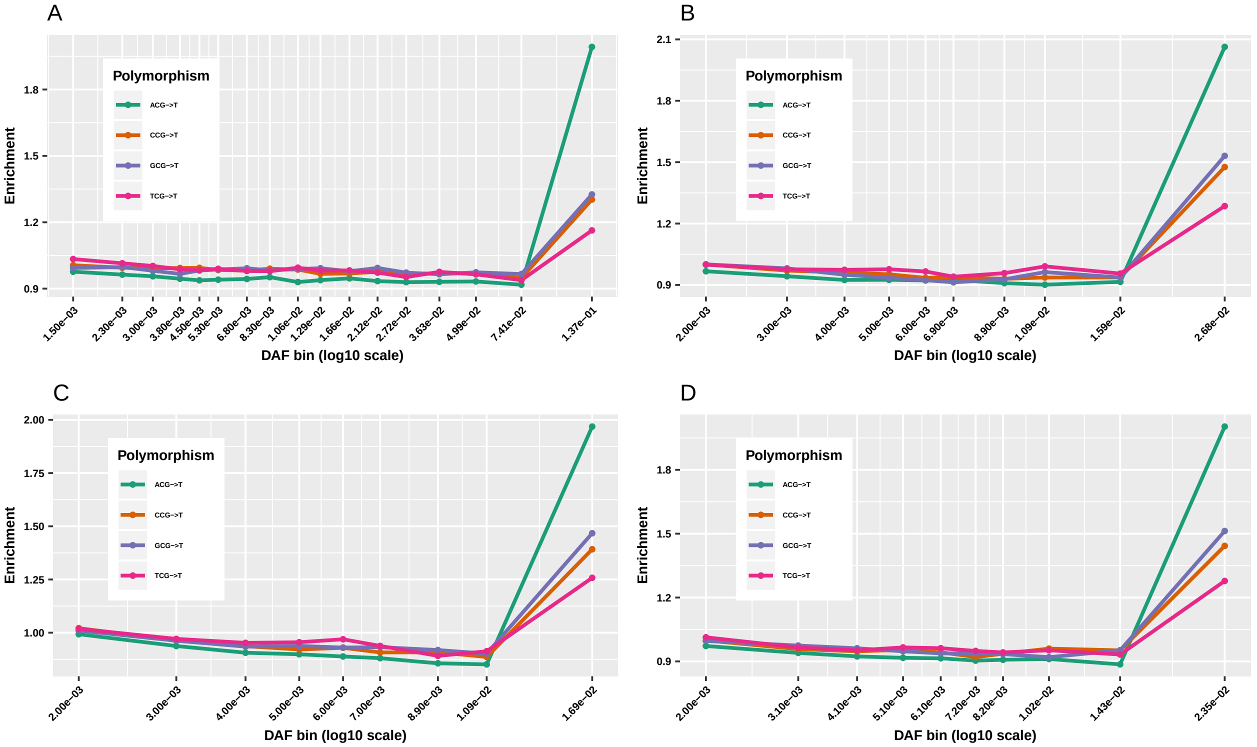

Supplementary Figure 19: DAF enrichment for singature #4 in (A) Africa, (B) East Asia, (C) Europe, (D) South Asia.
